# Synthesis, In Silico Studies, and Antioxidant and Tyrosinase Inhibitory Potential of 2-(Substituted Phenyl) Thiazolidine-4-Carboxamide Derivatives

**DOI:** 10.3390/ph16060835

**Published:** 2023-06-02

**Authors:** Muhammad Kazim Zargaham, Madiha Ahmed, Nosheen Akhtar, Zaman Ashraf, Mostafa A. Abdel-Maksoud, Mohammed Aufy, Humaira Nadeem

**Affiliations:** 1 Riphah Institute of Pharmaceutical Sciences, Riphah International University, Islamabad 04405, Pakistan; 2 Shifa College of Pharmaceutical Sciences, Shifa Tameer-e-Millat University, Islamabad 44000, Pakistan; 3 Department of Biological Sciences, National University of Medical Sciences, Rawalpindi 43600, Pakistan; 4 Department of Chemistry, Allama Iqbal Open University, Islamabad 44310, Pakistan; 5 Department of Botany and Microbiology, College of Science, King Saud University, Riyadh 11451, Saudi Arabia; 6 Department of Pharmaceutical Sciences, Division of Pharmacology and Toxicology, University of Vienna, A-1090 Vienna, Austria

**Keywords:** thiazolidine, tyrosinase inhibitors, mushroom tyrosinase, thiazolidine-4-carboxamide, molecular docking

## Abstract

Heterocyclic nuclei have shown a wide variety of biological activities, highlighting their importance in drug discovery. Derivatives of 2,4-subsituted thiazolidine have a structural similarity with the substrates of tyrosinase enzymes. Hence, they can be used as an inhibitor to compete against tyrosine in the biosynthesis of melanin. This study is focused on design, synthesis, biological activities, and in silico studies of thiazolidine derivatives substituted at positions 2 and 4. The synthesized compounds were evaluated to determine the antioxidant activity and tyrosine inhibitory potential using mushroom tyrosinase. The most potent tyrosinase enzyme inhibitor was compound **3c** having IC_50_ value 16.5 ± 0.37 µM, whereas compound **3d** showed maximum antioxidant activity in a DPPH free radical scavenging assay (IC_50_ = 18.17 µg/mL). Molecular docking studies were conducted using mushroom tyrosinase (PDB ID: 2Y9X) to analyze binding affinities and binding interactions of the protein–ligand complex. Docking results indicated that hydrogen bonds and hydrophobic interactions were mainly involved in the ligand and protein complex. The highest binding affinity was found to be −8.4 Kcal/mol. These results suggest that thiazolidine-4-carboxamide derivatives could serve as lead molecules for development of novel potential tyrosinase inhibitors.

## 1. Introduction

Thiazolidine is a heterocyclic organic compound at position 1 with a thioether group and position 3 with an amine group. It is a five-membered saturated ring with the molecular formula of (CH_2_)_3_(NH)S. Schubert first reported the thiazolidine carboxylic acid formation as a result of the reaction of cysteine with different aldehydes [1,2]. Thiazolidine nuclei can be synthesized using various methods, as summarized in a study published in 2020 [3].

In mushrooms and vertebrates, melanin biosynthesis is catalyzed by a copper-containing enzyme, tyrosinase [4]. Tyrosinase obtained from *Agaricus bisporus* has shown structural similarity to mammalian tyrosinase [5,6]. This structural similarity has helped researchers conduct melanogenic studies using mushroom tyrosinase. Kojic acid, a reversible inhibitor of the tyrosinase enzyme, interacts with the binding pocket without any conformational changes in the protein. Major reported amino acids present in the active site are Val283, His263, and Phe264. Other amino residues of the active side include His244, Glu256, Asn260, and residues 279–282 and Ala286 [7,8,9].

The tyrosinase enzyme is an important enzyme responsible for the attachment of marine organisms to their substrates as well as the insect molting process. In humans, it has been found to be linked with the pathogenesis of certain neurodegenerative diseases, including Parkinson’s disease [10]. The imperative function of this enzyme is melanin biosynthesis. It activates this pathway by catalyzing two steps, indicating its importance in melanin formation. Initially, tyrosine is converted to 3,4-dihydroxyphenylalanine (l-DOPA) by hydroxylation. In the second step, l-DOPA is converted to o-dopaquinone through oxidation [11,12]. Melanin is one of the most widely distributed pigments in animals, plants, fungi, and bacteria. It is responsible for pigmentation and color patterns on the skin of mammalians and protects them from the harmful effects of UV radiation [13]. Melanin deposits have also been found in the brain, and it is interesting to know that excessive levels of tyrosine cause the accumulation of epidermal melanin which can result in sites of actinic damage, age spots, melisma, and malignant melanoma [14]. Different strategies have been designed to address this issue by reducing melanin hyperpigmentation, which includes autophagy induction, by disrupting the transfer of melanosomes from melanocytes to keratinocytes by inducing melanosome dematuration [15]. Another aspect is that the formation of melanin beneath the skin is attributed to excessive free radicals that progress through UV radiation. The disruption of this mechanism through the inhibitory action of antioxidant compounds is a major strategy for the regulation of melanin formation [16]. The tyrosinase enzyme also catalyzes the hydroxylation of monophenols to catechols and subsequent oxidation to o-quinones using oxygen. The success of these strategies is accompanied by side effects, which include redness, crusting, swelling, itching, stinging, burning, and unusual discoloration [17]. Inhibitors are usually developed to compete with tyrosine and l-DOPA in the substrate-binding region of the tyrosinase enzyme. The side effects associated with the existing therapeutic regimens have persuaded the researchers to identify alternative strategies including the identification of novel tyrosinase enzyme inhibitors to facilitate the inhibition of melanin formation. Despite the discovery of various semisynthetic and synthetic inhibitors that are used against hyperpigmentation, there is still a need for safer and more effective tyrosinase inhibitors [18,19].

Different modifications have been made to the thiazolidine nucleus at position 2 to achieve inhibitory activity against the tyrosinase enzyme. Young Mi Ha et al. synthesized thiazolidine-4-carboxylic acid analogs with substitution at carbon number 2 as novel tyrosinase inhibitors. The structure of derivatives was rationalized using structural features of a potent inhibitor of catechol oxidase enzyme, N-phenylthiourea, along with the substrate and product of tyrosinase [20]. Some other compounds containing thiazolidine-like nuclei also possess tyrosinase inhibitory activity. These compounds have benzothiazole, benzothiazoline, benzimidazole, and thiazoline nuclei instead of thiazolidine [17,18].

The thiazolidine nucleus can be modified in different ways to obtain a wide variety of derivatives. Although many derivatives of this nucleus have been synthesized and evaluated for different biological activities, which highlight its importance [21,22], new derivatives can be designed having potential biological activities. Thiazolidine-4-carboxylic derivatives reported so far have been modified at position 2 with a free carboxylic acid group at position 4. Position 4 can be further explored for designing compounds having potential biological effects. The main aim of this research was to design and synthesize a range of 2,4-substituted thiazolidine derivatives with potential tyrosinase inhibition by modifying the 4-carboxylic acid group. The antioxidant and tyrosinase inhibitory potential of the compounds synthesized was evaluated. Molecular docking evaluation was conducted to computationally validate the findings of this study. The rational for selecting tyrosinase activity was the structural similarity of 2-(substituted phenyl) thiazolidine-4-carboxamide derivatives with tyrosine, DOPA, and N-phenylthiourea. N-phenylthiourea has shown inhibitory potential against catechol oxidase enzyme, which belongs to the same type of enzymes as tyrosinase [23]. Since reactive oxygen species can increase tyrosinase expression [24], we investigated the antioxidant potential of the synthesized compounds. Compounds having antioxidant activity may exhibit enhanced inhibitory effects on melanin synthesis.

## 2. Results and Discussion

### 2.1. Chemistry

**Scheme 1:**
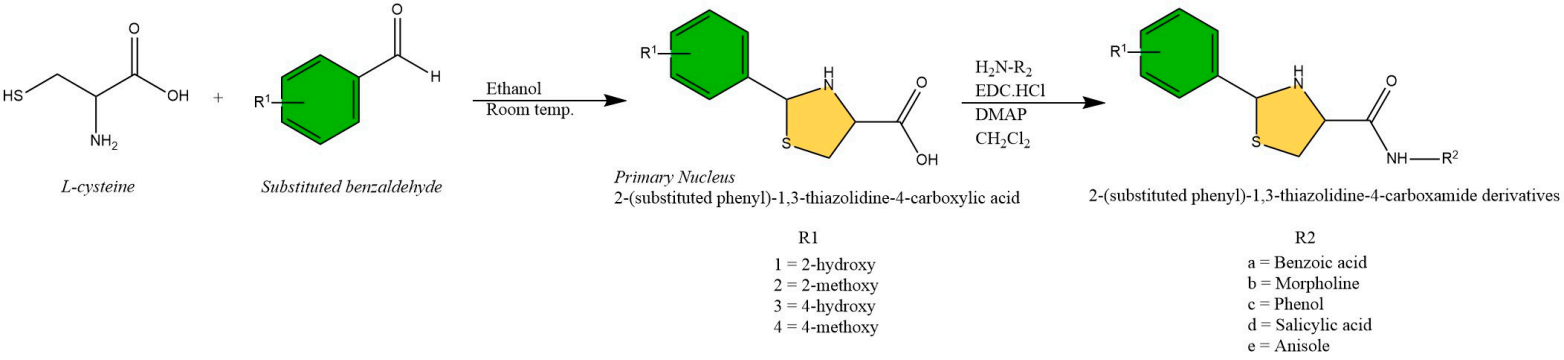
Synthetic route to 2-(substituted phenyl)-1,3-thiazolidine-4-carboxamide.

Scheme 1 represents the two steps involved in the synthesis of thiazolidine-4-carboxamide derivatives. In the first step, l-cysteine hydrochloride monohydrate was reacted with substituted benzaldehyde in the presence of sodium bicarbonate. This led to the formation of 2-(substituted phenyl) thiazolidine-4-carboxylic acids. The reaction was processed at room temperature. White-colored precipitates obtained were washed with water and ethanol. This was followed by drying in a vacuum desiccator to obtain the pure product. The reaction time and conditions were consistent with the previously reported schemes [20,25]. The reaction time ranged from 3 h to 12 h. The product formation was confirmed by comparing the melting points reported in a previous study [20]. The yield of the substituted thiazolidine nuclei (**1**–**4**) varied from 40 to 90%.

**Figure 1:**
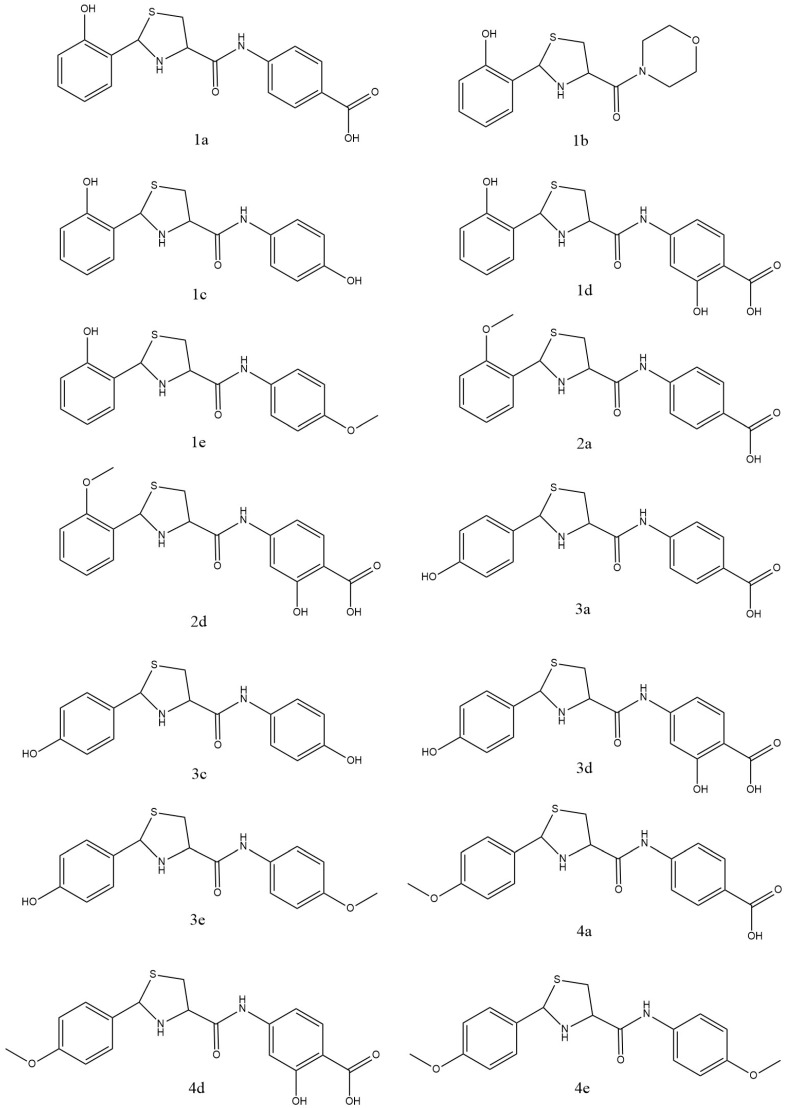
Structures of the synthesized compounds.

These nuclei (**1–4**) were used further to synthesize the carboxamide derivatives. Nuclei from the first step, 2-(substituted phenyl) thiazolidine-4-carboxylic acids (**1**–**4**), were reacted with an amine in the presence of N-(3-Dimethylaminopropyl)-N′-ethylcarbodiimide hydrochloride (EDC.HCl) and 4-Dimethylaminopyridine (DMAP) in dichloromethane. Stirring was carried out at room temperature for a time ranging from 4–24 h [26]. A gel-like product was obtained on reaction completion which was washed with methanol and water to obtain the product in powder form. The amines used were p-amino benzoic acid, morpholine, p-amino phenol, p-amino salicylic acid, and p-anisidine. As a result, five carboxamide derivatives were obtained numbered as **a, b, c, d,** and **e**, respectively. Nuclei with 2-methoxyphenyl (2) and 4-methoxyphenyl (4) gave a lesser yield with a longer reaction time owing to the steric hindrance, leading to the synthesis of limited derivatives, i.e., two for 2-methoxyphenyl (**2a** and **2d**) and three derivatives in the case of 4-methoxyphenyl (**4a**, **4d** and **4e**). The yield of the final products varied from 20–72% (Table 1). In total, 14 derivatives were synthesized based on the four substituted thiazolidine nuclei and five amines. The purity of the synthesized compounds was confirmed through TLC. The chemo-informatics analysis of synthesized derivatives is presented in Table S1. Figure 1 represents the structures of all the synthesized compounds.

Derivatives synthesized were characterized using spectral data from FTIR, ^1^H-NMR, and ^13^C-NMR (Figures S1–S8). The expected stretches were observed in the spectral data of FTIR. Further, chemical shift values from proton and carbon NMR were used for structural confirmation of the planned derivatives. The chemical shift values of thiazolidine protons were compared with the NMR results of the previously reported thiazolidine-4-carboxylic acid derivatives [20]. Observed protons of the thiazolidine nucleus included 2-H, 4-H, and 5-H_a_, H_b_. The proton of amine (NH) mostly gives a weak signal and is usually not reported. A peak of the proton of carboxamide was observed in all the compounds, which confirmed the structure of 4-carboxamide derivatives of the thiazolidine nuclei. A singlet was observed between the ranges of 8.112–8.998 ppm.

### 2.2. Biological Evaluation

#### 2.2.1. Determination of Antioxidant Activity

**Figure 2:**
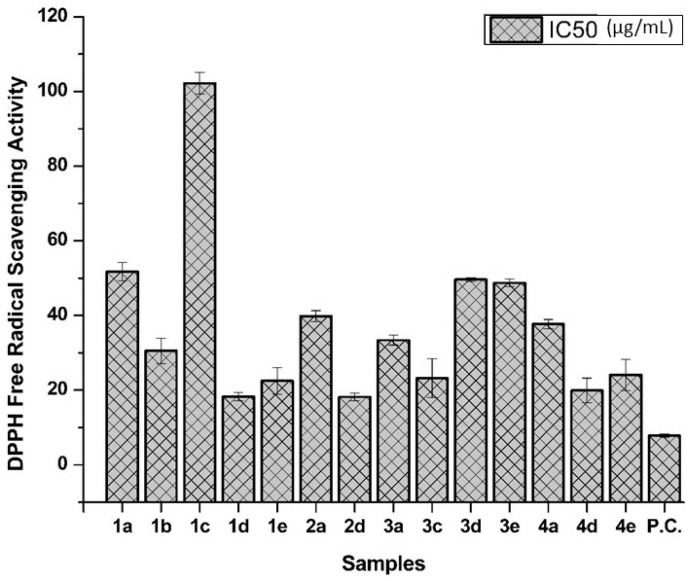
The IC_50_ values of synthesized compounds in the DPPH free radical scavenging assay. The results are presented as mean ± SD. P.C. stands for positive control (ascorbic acid).

The 2,2-Diphenyl-1-picrylhydrazyl (DPPH) radical was used to evaluate the antioxidant potential of the synthesized compounds. Different concentrations of the samples were employed in the subject assay. The maximum antioxidant potential was shown by the compound **2d** (IC_50_ 18.17 ± 1.0 µg/mL) in comparison with the antioxidant activity of the positive control, ascorbic acid (IC_50_ 7.83 ± 0.5 µg/mL) (Figure 2). The compound **1d** also showed significant antioxidant potential with an IC_50_ value of 18.27 ± 1.1 µg/mL. The principle of this assay is related to the conversion of the purple-colored free radical into the pale-yellow-colored reduced form, owing to the action of a proton donating or antioxidant agent [27]. The compound with the most prominent activity (**2d**) possessed hydroxyls at position 3 of the ring containing the carboxamide group, whereas the methoxy group was attached to position 2 of the benzene ring. Alternatively, compound **1d** also acquired hydroxy groups on both mentioned positions. These groups are categorized as strong proton donors which tend to participate in the reduction in DPPH free radicals by donating protons.

#### 2.2.2. Tyrosinase Inhibition Studies

An evaluation of the inhibitory effect on the tyrosinase enzyme was conducted on mushroom tyrosinase. The results were compared with the competitive inhibitor of the tyrosinase enzyme, kojic acid (Table 2). Among the synthesized derivatives, the maximum inhibitory potential was observed in the case of compound **3c** (IC_50_ 16.5 ± 0.37 µM), compared with the IC_50_ value of the standard, kojic acid (15.9 ± 2.5 µM). Compounds **4e**, **3e**, **2d,** and **2a** also exhibited moderate activity. Compounds **3c**, **3e,** and **4e** exhibited the presence of oxygen atoms at the para-position on both benzene rings in the form of hydroxyl and methoxy groups. The presence of oxygen atoms at these positions could be interacting with key amino acids and the copper ions of the enzyme, resulting in higher inhibitory potential. The remaining derivatives showed insignificant activity with values of IC_50_ varying between 98.5 ± 10.6 and 256 ± 16.3 µM.

### 2.3. Molecular Docking

**Figure 3:**
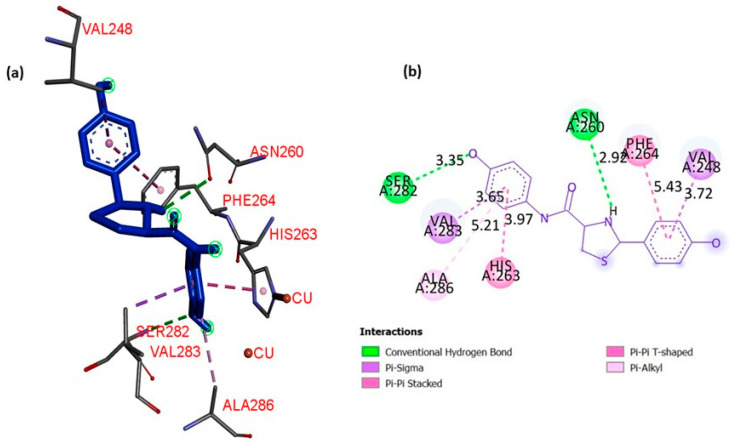
Binding interactions between derivative **3c** and mushroom tyrosinase active site (PDB-ID 2Y9X). (**a**) Binding conformation of compound **3c** in 3D along with the binding interaction of the protein–ligand complex. (**b**) Interaction patterns in 2D indicating the type and distance of the interaction. Legend indicates various types of binding interactions present.

All synthesized compounds exhibited binding affinities higher than the reference compound, kojic acid. Important amino acids of the active site, His244, Glu256, Asn260, and residues 279–282 and Ala286, were involved in the ligand–protein interactions (Table 3) (Figure S9–S23). The distance of the binding interactions varied from 1.93 Å to 5.70 Å. The highest binding affinity was exhibited by compound **3c** (−8.4 Kcal/mol) (Figure 3), while the lowest binding affinity was exhibited by compounds **1e** and **3e** (−6.9 Kcal/mol). Kojic acid exhibited a binding affinity of −5.5 Kcal/mol. Two derivatives of the 4-hydroxy primary nucleus, **3c** and **3e**, with lower IC_50_, represent the importance of the hydroxyl group at the fourth position. This activity was decreased by the modification of the carboxylic acid group towards the carboxamide end (compound **3a**), probably owing to the bulky nature of the attached group. A similar pattern was observed in compounds **1a** and **1d**, owing to the carboxylic acid group and higher IC_50_ values. Derivatives with the hydroxyl group at the second position (**1a**–**1e**) exhibited higher IC_50_ values. This hydroxyl group can form intramolecular hydrogen bonding with the sulfur atom of the thiazolidine ring, resulting in poor interaction with the target enzyme. This observation was supported by the results of derivatives with the 2-methoxy group (**2a** and **2d**). The 2-methoxy derivatives showed lower IC_50_ values in comparison with the 2-hydroxy derivatives. All the methoxy derivatives (**2a**, **2d**, and **4e**) exhibited lower IC_50_ values. Some thiazolidine derivatives have been previously synthesized, which also showed tyrosinase inhibitory potential supporting the rational of this study for selecting thiazolidine nucleus [20,28]. Thiazolidine derivatives synthesized in this study are novel through the modification of the carboxamide group at the fourth position of the nucleus.

## 3. Materials and Methods

### 3.1. Instruments and Chemicals

Required chemicals and solvents were procured from Sigma-Aldrich (St. Louis, MO, USA), Merck (Kenilworth, NJ, USA), and Honeywell (Charlotte, NC, USA) with no further purification before their use. Spectrophotometric and elemental analysis was used for the characterization of the synthesized derivatives. Functional group detection was performed with a Bruker ALPHA FTIR spectrometer (Billerica, MA, USA), and structural analysis was conducted with ^1^HNMR and ^13^C NMR using a Bruker AM300 spectrophotometer (Billerica, MA, USA) with DMSO as the solvent. Melting points were also recorded with the help of the Gallenkamp melting point apparatus. Mushroom tyrosinase (EC 1.14.18.1) was purchased from Sigma-Aldrich, (St. Louis, MO, USA).

### 3.2. General Procedure for the Synthesis of the Thiazolidine Nucleus

A previously reported scheme was used for the synthesis of the thiazolidine nucleus [20,25]. A total of four 2-(substituted phenyl) thiazolidine-4-carboxylic acid derivatives were synthesized using differently substituted benzaldehydes. l-cysteine hydrochloride monohydrate (6.2 mmol) was added to 100 mL of water. Then, sodium bicarbonate (6.2 mmol) was added to the mixture with stirring. A solution of respective benzaldehyde (6.2 mmol) in ethanol (100 mL) was prepared separately and added to the reaction mixture. The mixture was stirred for 2.5 h at room temperature. The precipitates formed were collected through filtration and washed with cooled water and ethanol.

#### 3.2.1. 2-(2-Hydroxyphenyl)-1,3-thiazolidine-4-carboxylic Acid (**1**)

White solid; mp_(observed)_: 173–175 °C; mp_(lit)_: 173.2–175.1 °C; yield: 90%; ^1^H-NMR (DMSO, δ ppm): 3.009 (d, J = 14.30 Hz, 1H, 5-H_a_), 3.228 (d, J = 14.30 Hz, 1H, 5-H_b_), 4.235 (dd, ^1^J = 9.37 Hz, ^2^J = 5.64 Hz, 1H, 4-H), 5.699 (s, 1H, 2-H), 6.525–7.625 (m, 4H, Ar-H), 9.868 (s, 1H, Ar-2′OH).

#### 3.2.2. 2-(2-Methoxyphenyl)-1,3-thiazolidine-4-carboxylic Acid (**2**)

White solid; m.p._(observed)_: 137–139 °C; m.p._(lit)_: 137.7–139.2 °C; yield: 40%; ^1^H-NMR (DMSO, δ ppm): 3.495 (d, J = 14.31, 1H, 5-H_a_), 3.551 (d, J = 14.31, 1H, 5-H_b_), 3.795 (s, 3H, Ar-2′O CH_3_), 3.919 (dd, ^1^J = 9.52, ^2^J = 5.39, 1H, 4-H), 5.135 (s, 1H, 2-H), 6.953–8.049 (m, 4H, Ar-H).

#### 3.2.3. 2-(4-Hydroxyphenyl)-1,3-thiazolidine-4-carboxylic Acid (**3**)

White solid; m.p._(observed)_: 161–164 °C; m.p._(lit)_: 161.8–164.4 °C; yield: 55%; ^1^H-NMR (DMSO, δ ppm): 3.404 (d, J = 14.29, 1H, 5-H_a_), 3.621 (d, J = 14.29, 1H, 5-H_b_), 3.693 (dd, ^1^J = 9.37, ^2^J = 5.64, 1H, 4-H), 5.236 (s, 1H, 2-H), 6.653–8.049 (m, 4H, Ar-H), 9.563 (s, 1H, Ar-4′OH).

#### 3.2.4. 2-(4-Methoxyphenyl)-1,3-thiazolidine-4-carboxylic Acid (**4**)

White solid; m.p._(observed)_: 157–158 °C; m.p._(lit)_: 157.6–158.2 °C; yield: 45%; ^1^H-NMR (DMSO, δ ppm): 3.494 (d, J = 14.31, 1H, 5-H_a_), 3.549 (d, J = 14.31, 1H, 5-H_b_), 3.747 (s, 3H, Ar-4′OCH_3_), 3.887 (dd, ^1^J = 9.52, ^2^J = 5.39, 1H, 4-H), 5.147 (s, 1H, 2-H), 6.895–8.049 (m, 4H, Ar-H).

### 3.3. General Scheme for the Synthesis of Carboxamide Derivatives from Thiazolidine Nucleus

Carboxamide derivatives were synthesized from the thiazolidine nucleus by reacting with various amines [26]. 2-(substituted phenyl) thiazolidine-4-carboxylic acid (0.98 mmol), amine (1.12 mmol), DMAP (1.09 mmol), and EDC.HCl (1.08 mmol) were mixed in dichloromethane (2 mL). Then, stirring for 4 h was carried out at room temperature. The product was obtained in the form of a hard gel. This gel-type product was washed with methanol and water to obtain a purified product in the form of a powder.

#### 3.3.1. 4-[2-(2-Hydroxyphenyl)-1,3-thiazolidine-4-carbonyl]-aminobenzoic Acid (**1a**)

Yellow solid; yield: 54%; m.p.: 135–136 °C; IR cm^−1^: 3500 (O-H), 3100 (N-H stretch), 1600 (C=O), 1500 (N-H bend), 1470 (C=C); ^1^H-NMR (DMSO, δ ppm): 3.009 (d, J = 14.30 Hz, 1H, 5-H_a_), 3.228 (d, J = 14.30 Hz, 1H, 5-H_b_), 4.235 (dd, ^1^J = 9.37 Hz, ^2^J = 5.64 Hz, 1H, 4-H), 5.699 (s, 1H, 2-H), 6.525–7.625 (m, 8H, Ar-H), 8.141 (s, 1H, amide-NH), 9.868 (s, 1H, Ar-2′OH), 11.295 (s, 1H, 4″COOH); ^13^C-NMR (DMSO, 100 MHz, δ ppm): 172.4 (CO-NH), 168.2 (COOH), 161.0 (C-2′), 136.4 (C-1′), 132.5 (C-3″, C-5″), 130.2 (C-4′), 127.4 (C-5′), 126.5 (C-1′), 126.0 (C-6′), 125.7 (C-4″), 119.0 (C-2″, C-6″), 118.2 (C-3′), 66.2 (C-2), 56.2 (C-4), 32.4 (C-5); Anal. Calcd. for C_17_H_16_N_2_O_4_S: C, 58.88; H, 3.95; N, 7.92; O, 17.96; S, 10.201. Found: C, 58.15; H, 3.30; N, 7.22; O, 18.40; S, 9.25.

#### 3.3.2. [2-(2-Hydroxyphenyl)-1,3-thiazolidin-4-yl](morpholin-4-yl)-methanone (**1b**)

Orange solid; yield: 68%; m.p.: 128–129 °C; IR cm^−1^: 3600 (O-H), 3120 (N-H stretch), 1640 (C=O), 1590 (N-H bend), 1448 (C=C); ^1^H-NMR (DMSO, δ ppm): 3.393 (d, J = 14.34, 1H, 5-H_a_), 3.598 (d, J = 14.34, 1H, 5-H_b_), 3.624 (m, 8H, CH_2_), 3.712 (dd, ^1^J =9.37, ^2^J = 5.64, 1H, 4-H), 5.436 (s, 1H, 2-H), 6.658–7.398 (m, 8H, Ar-H); ^13^C-NMR (DMSO, 100 MHz, δ ppm): 170.2 (CO-NH), 160.5 (C-2′), 130.2 (C-4′), 129.5 (C-5′), 129.0 (C-1′), 128.6 (C-6′), 117.2 (C-3′), 66.8 (C-3″, C-5″), 64.8 (C-2), 57.4 (C-4), 44.5 (C-2″, C-6″), 31.6 (C-5); Anal. Calcd. for C_14_H_18_N_2_O_3_S: C, 57.12; H, 5.95; N, 8.85; O, 16.31; S, 9.74. Found: C, 56.95; H, 6.05; N, 8.45; O, 16.35; S, 9.80.

#### 3.3.3. 2-(2-Hydroxyphenyl)-*N*-(4-hydroxyphenyl)-1,3-thiazolidine-4-carboxamide (**1c**)

Black solid; yield: 45%; m.p.: 109–111 °C; IR cm^−1^: 3530 (O-H), 3400 (N-H stretch), 1650(C=O), 1500 (N-H bend), 1410 (C=C); ^1^H-NMR (DMSO, δ ppm): 3.408 (d, J = 14.31, 1H, 5-H_a_), 3.610 (d, J = 14.31, 1H, 5-H_b_), 3.717 (dd, ^1^J = 9.37, ^2^J = 5.64, 1H, 4-H), 5.437 (s, 1H, 2-H), 6.658–7.398 (m, 8H, Ar-H), 8.518 (s, 1H, amide-NH), 9.218 (s, 1H, Ar-2′OH), 9.965 (s, 1H, Ar-4″OH); ^13^C-NMR (DMSO, 100 MHz, δ ppm): 174.8 (CO-NH), 162.0 (C-2′), 158.4 (C-4″), 137.8 (C-1″), 130.4 (C-4′), 128.4 (C-5′), 127.0 (C-1′), 125.8 (C-6′), 120.5 (C-5″, C-6″), 116.5 (C-3′), 113.2 (C-3″, C-5″), 66.2 (C-2), 55.4 (C-4), 30.6 (C-5). Anal. Calcd. for C_16_H_16_N_2_O_3_S: C, 59.85; H, 4.95; N, 9.12; O, 15.17; S, 9.03. Found: C, 59.70; H, 4.02; N, 9.50; O, 15.20; S, 10.10.

#### 3.3.4. 2-Hydroxy-4-[2-(2-hydroxyphenyl)-1,3-thiazolidine-4-carbonyl]-aminobenzoic Acid (**1d**)

Yellow solid; yield: 68%; m.p.: 134–135 °C; IR cm^−1^: 3530 (O-H), 3150 (N-H stretch), 1643 (C=O), 1590 (N-H bend), 1400 (C=C); ^1^H-NMR (DMSO, δ ppm): 3.406 (d, J = 14.29, 1H, 5-H_a_), 3.623 (d, J = 14.29, 1H, 5-H_b_), 3.758 (dd, ^1^J = 9.37, ^2^J = 5.64, 1H, 4-H), 5.437 (s, 1H, 2-H), 6.308–8.023 (m, 8H, Ar-H), 8.123 (s, 1H, amide-NH), 9.225 (s, 1H, Ar-2′OH), 9.995 (s, 1H, Ar-3″OH), 11.598 (s, 1H, 4″-COOH); ^13^C-NMR (DMSO, 100 MHz, δ ppm): 173.5 (CO-NH),170.8 (COOH), 165.3 (C-3″), 161.0 (C-2′), 140.2 (C-1″), 133.4 (C-5″), 129.9 (C-4′), 128.8 (C-5′), 128.0 (C-1′), 126.0 (C-6′), 118.5 (C-6″), 115.8 (C-3′), 111.8 (C-4″), 101.1 (C-2″), 66.4 (C-2), 55.2 (C-4), 31.1 (C-5); Anal. Calcd. for C_17_H_16_N_2_O_5_S: C, 57.22; H, 5.04; N, 8.12; O, 21.10; S, 9.15. Found: C, 57.60; H, 5.45; N, 8.65; O, 21.18; S, 9.83.

#### 3.3.5. 2-(2-Hydroxyphenyl)-*N*-(4-methoxyphenyl)-1,3-thiazolidine-4-carboxamide (**1e**)

Yellow solid; yield: 60%; m.p.: 83–84 °C; IR cm^−1^: 3600 (O-H), 3100 (N-H stretch), 1670 (C=O), 1490 (N-H bend), 1450 (C=C); ^1^H-NMR (DMSO, δ ppm): 3.152 (d, J = 14.04, 1H, 5-H_a_), 3.324 (d, J = 14.04, 1H, 5-H_b_), 3.797(s, 3H, 4″-OCH_3_), 4.358 (dd, ^1^J = 9.52, ^2^J = 5.39, 1H, 4-H), 5.298 (s, 1H, 2-H), 6.935–7.633 (m, 8H, Ar-H), 8.425 (s, 1H, amide-NH), 9.525 (s, 1H, Ar-2′OH); ^13^C-NMR (DMSO, 100 MHz, δ ppm): 173.6 (CO-NH), 162.0 (C-2′), 155.8 (C-4″), 138.4 (C-1″), 129.2 (C-4′), 128.4 (C-5′), 128.0 (C-1′), 127.2 (C-6′), 121.5 (C-2″. C-6″), 116.9 (C-3′), 113.5 (C-3″, C-5″), 66.5 (C-2), 56.6 (C-4), 55.0 (4′-OCH3), 31.8 (C-5). Anal. Calcd. for C_17_H_18_N_2_O_3_S: C, 61.80; H, 4.95; N, 7.98; O, 14.53; S, 10.03. Found: C, 61.75; H, 4.45; N, 7.42; O, 14.48; S, 10.60.

#### 3.3.6. 4-[2-(2-Methoxyphenyl)-1,3-thiazolidine-4-carbonyl]-aminobenzoic Acid (**2a**)

White solid; yield: 20%; m.p.: 116–117 °C; IR cm^−1^: 3500 (O-H), 3220 (N-H stretch), 1600 (C=O), 1498 (N-H bend), 1380 (C=C); ^1^H-NMR (DMSO, δ ppm): 3.495 (d, J = 14.31, 1H, 5-H_a_), 3.551 (d, J = 14.31, 1H, 5-H_b_), 3.795 (s, 3H, Ar-2′O CH_3_), 3.919 (dd, ^1^J = 9.52, ^2^J = 5.39, 1H, 4-H), 5.135 (s, 1H, 2-H), 6.953–8.049 (m, 8H, Ar-H), 8.235 (s, 1H, amide-NH), 11.285 (s, 1H, 4″-COOH); ^13^C-NMR (DMSO, 100 MHz, δ ppm): 170.2 (CO-NH), 165.1 (COOH), 155.3 (C-2′), 138.4 (C-1″), 131.5 (C-3″, C-5″), 129.4 (C-4′), 128.5 (C-5′), 128.0 (C-1′), 127.8 (C-6′), 127.0 (C-4″), 117.9 (C-2″, C-6″), 114.8 (C-3′), 66.2 (C-2), 56.8 (C-4), 55.0 (2′-OCH3), 30.9 (C-5). Anal. Calcd. for C_18_H_18_N_2_O_4_S: C, 61.14; H, 4.92; N, 8.03; O, 17.86; S, 9.02. Found: C, 61.20; H, 4.95; N, 8.45; O, 17.90; S, 8.88.

#### 3.3.7. 2-Hydroxy-4-[2-(2-methoxyphenyl)-1,3-thiazolidine-4-carbonyl]-aminobenzoic Acid (**2d**)

White solid; yield: 25%; m.p.: 112–113 °C; IR cm^−1^: 3510 (O-H), 3300 (N-H stretch), 1660 (C=O), 1489 (N-H bend), 1386 (C=C); ^1^H-NMR (DMSO, δ ppm): 3.496 (d, J = 14.31, 1H, 5-H_a_), 3.551 (d, J = 14.31, 1H, 5-H_b_), 3.795 (s, 3H, Ar-2′O CH_3_), 3.888 (dd, ^1^J = 9.52, ^2^J = 5.39, 1H, 4-H), 5.135 (s, 1H, 2-H), 6.953–7.395 (m, 8H, Ar-H), 8.586 (s, 1H, amide-NH), 9.228 (s, 1H, Ar-3″OH), 11.298 (s, 1H, 4″-COOH); ^13^C-NMR (DMSO, 100 MHz, δ ppm): 174.2 (CO-NH), 172.3 (COOH), 162.3 (C-3″), 158.3 (C-2′), 140.3 (C-1″), 132.3 (C-5″), 130.4 (C-4′), 128.5 (C-5′), 128.0 (C-1′), 126.8 (C-6′), 118.9 (C-6″), 114.5 (C-3′), 110.2 (C-4″), 100.4 (C-2″), 64.2 (C-2), 56.5 (C-4), 54.0 (2′-OCH3), 31.2 (C-5); Anal. Calcd. for C_18_H_18_N_2_O_5_S: C, 60.12; H, 5.02; N, 8.22; O, 20.13; S, 9.42. Found: C, 60.22; H, 5.22; N, 8.42; O, 21.24; S, 8.42.

#### 3.3.8. 4-[2-(4-Hydroxyphenyl)-1,3-thiazolidine-4-carbonyl]-aminobenzoic Acid (**3a**)

Yellow solid; yield: 72%; m.p.: 164–166 °C; IR cm^−1^: 3550 (O-H), 3000 (N-H stretch), 1597 (C=O), 1508 (N-H bend), 1450 (C=C); ^1^H-NMR (DMSO, δ ppm): 3.404 (d, J = 14.29, 1H, 5-H_a_), 3.621 (d, J = 14.29, 1H, 5-H_b_), 3.693 (dd, ^1^J = 9.37, ^2^J = 5.64, 1H, 4-H), 5.236 (s, 1H, 2-H), 6.653–8.049 (m, 8H, Ar-H), 8.998 (s, 1H, amide-NH), 9.563 (s, 1H, Ar-4′OH), 12.004 (s, 1H, 4″COOH); ^13^C-NMR (DMSO, 100 MHz, δ ppm): 173.7 (CO-NH), 164.5 (COOH), 159.4 (C-4′), 138.4 (C-1″), 135.1 (C-1′), 131.2 (C-3″, C-5″), 127.2 (C-2′, C-6′), 125.1 (C-4″), 117.8 (C-2″, C-6”), 114.7 (C-3′, C-5′), 63.2 (C-2), 54.6 (C-4), 32.6 (C-5); Anal. Calcd. for C_17_H_16_N_2_O_4_S: C, 60.12; H, 5.14; N, 7.95; O, 18.58; S, 8.92. Found: C, 60.10; H, 5.40; N, 7.92; O, 18.40; S, 8.45.

#### 3.3.9. *N*,2-Bis(4-hydroxyphenyl)-1,3-thiazolidine-4-carboxamide (**3c**)

Black solid; yield: 68%; m.p.: 174–176 °C; IR cm^−1^: 3580 (O-H), 3050 (N-H stretch), 1640 (C=O), 1512 (N-H bend), 1400 (C=C); ^1^H-NMR (DMSO, δ ppm): 3.408 (d, J = 14.30, 1H, 5-H_a_), 3.615 (d, J = 14.30, 1H, 5-H_b_), 3.692 (dd, ^1^J = 9.37, ^2^J = 5.64, 1H, 4-H), 5.326 (s, 1H, 2-H), 6.653–7.278 (m, 8H, Ar-H), 8.112 (s, 1H, amide-NH), 9.158 (s, 1H, Ar-4′OH), 9.998 (s, 1H, 4″OH); ^13^C-NMR (DMSO, 100 MHz, δ ppm): 170.2 (CO-NH), 157.5 (C-4′), 138.4 (C-1″), 134.2 (C-1′), 128.7 (C-2′, C-6′), 120.8 (C-2″, C-6″), 116.2 (C-3′, C5′), 114.2 (C-3″, C-5″), 64.3 (C-2), 55.6 (C-4), 32.4 (C-5); Anal. Calcd. for C_16_H_16_N_2_O_3_S: C, 59.89; H, 4.92; N, 9.12; O, 15.17; S, 9.24. Found: C, 59.95; H, 4.98; N, 9.22; O, 15.03; S, 10.02.

#### 3.3.10. 2-Hydroxy-4-[2-(4-hydroxyphenyl)-1,3-thiazolidine-4-carbonyl]-aminobenzoic Acid (**3d**)

Yellow solid; yield: 68%; m.p.: 130–131 °C; IR cm^−1^: 3500 (O-H), 3100 (N-H stretch), 1600 (C=O), 1560 (N-H bend), 1450 (C=C); ^1^H-NMR (DMSO, δ ppm): 3.405 (d, J = 14.29, 1H, 5-H_a_), 3.622 (d, J = 14.29, 1H, 5-H_b_), 3.696 (dd, ^1^J = 9.37, ^2^J = 5.64, 1H, 4-H), 5.327 (s, 1H, 2-H), 6.307–8.023 (m, 8H, Ar-H), 8.534 (s, 1H, amide-NH), 9.125 (s, 1H, Ar-4′OH), 9.893 (s, 1H, Ar-3″OH), 11.518 (s, 1H, 4″COOH); ^13^C-NMR (DMSO, 100 MHz, δ ppm): 173.2 (CO-NH), 170.1 (COOH), 162.5 (C-3″), 158.4 (C-4′), 139.3 (C-1″), 135.8 (C-1′), 130.5 (C-5″), 128.8 (C-2′, C-6′), 118.0 (C-6″), 114.4 (C-3′, C-5′), 109.3 (C-4″), 101.2 (C-2″), 64.2 (C-2), 54.5 (C-4), 33.6 (C-5); Anal. Calcd. for C_17_H_16_N_2_O_5_S: C, 57.12; H, 5.15; N, 8.32; O, 21.95; S, 9.12. Found: C, 57.50; H, 5.35; N, 8.70; O, 21.80; S, 9.05.

#### 3.3.11. 2-(4-Hydroxyphenyl)-N-(4-methoxyphenyl)-1,3-thiazolidine-4-carboxamide (**3e**)

Brown solid; yield: 65%; m.p.: 115–117 °C; IR cm^−1^: 3500 (O-H), 3100 (N-H stretch), 1600 (C=O), 1550 (N-H bend), 1440 (C=C); ^1^H-NMR (DMSO, δ ppm): 3.445 (d, J = 14.33, 1H, 5-H_a_), 3.539 (d, J = 14.33, 1H, 5-H_b_), 3.715 (s, 3H, 4″OCH_3_), 3.825 (dd, ^1^J = 9.52, ^2^J = 5.39, 1H, 4-H), 5.154 (s, 1H, 2-H), 6.653–7.275 (m, 8H, Ar-H), 8.224 (s, 1H, amide-NH), 9.212 (s, 1H, Ar-4′OH); ^13^C-NMR (DMSO, 100 MHz, δ ppm): 172.2 (COOH), 157.7 (C-4″), 155.4 (C-4′), 137.8 (C-1″), 134.2 (C-1′), 128.8 (C-2′, C-6′), 121.5 (C-2″, C-6″), 118.4 (C3′, C-5′), 115.2 (C-3″, C-5″), 64.2 (C-2), 57.2 (C-4), 55.0 (4″-OCH3), 32.2 (C-5); Anal. Calcd. for C_17_H_18_N_2_O_3_S: C, 61.80; H, 4.49; N, 9.05; O, 14.53; S, 10.12. Found: C, 61.78; H, 4.30; N, 9.35; O, 14.22; S, 10.63.

#### 3.3.12. 4-[2-(4-Methoxyphenyl)-1,3-thiazolidine-4-carbonyl]-aminobenzoic Acid (**4a**)

Yellow solid; yield: 60%; m.p.: 94–95 °C; IR cm^−1^: 3610 (O-H), 3200 (N-H stretch), 1590 (C=O), 1550 (N-H bend), 1420 (C=C); ^1^H-NMR (DMSO, δ ppm): 3.494 (d, J = 14.31, 1H, 5-H_a_), 3.549 (d, J = 14.31, 1H, 5-H_b_), 3.747 (s, 3H, Ar-4′OCH_3_), 3.887 (dd, ^1^J = 9.52, ^2^J = 5.39, 1H, 4-H), 5.147 (s, 1H, 2-H), 6.895–8.049 (m, 8H, Ar-H), 8.152 (s, 1H, amide-NH), 11.892 (s, 1H, 4″COOH); ^13^C-NMR (DMSO, 100 MHz, δ ppm): 171.8 (CO-NH), 167.8 (COOH), 160.9 (C-4′), 136.2 (C-1″), 135.0 (C-1′), 131.4 (C-3″, C-5″), 129.4 (C-2′, C-6′), 128.2 (C-4″), 118.8 (C-2″, C-6″), 113.3 (C-3′, C-5′), 64.2 (C-2), 56.6 (4′-OCH3), 54.0 (C-4), 32.6 (C-5). Anal. Calcd. for, C_18_H_18_N_2_O_4_S: C, 59.92; H, 4.80; N, 8.13; O, 17.86; S, 9.21. Found: C, 59.75; H, 4.92; N, 8.66; O, 17.70; S, 8.84.

#### 3.3.13. 2-Hydroxy-4-[2-(4-methoxyphenyl)-1,3-thiazolidine-4-carbonyl]-aminobenzoic Acid (**4d**)

White solid; yield: 66%; m.p.: 109–110 °C; IR cm^−1^: 3600 (O-H), 3120 (N-H stretch), 1645 (C=O), 1511 (N-H bend), 1420 (C=C); ^1^H-NMR (DMSO, δ ppm): 3.461 (d, J = 14.31, 1H, 5-H_a_), 3.552 (d, J = 14.31, 1H, 5-H_b_), 3.744 (s, 3H, Ar-4′OCH_3_), 3.861 (dd, ^1^J = 9.52, ^2^J = 5.39, 1H, 4-H), 5.148 (s, 1H, 2-H), 6.307–8.023 (m, 8H, Ar-H), 8.212 (s, 1H, amide-NH), 9.518 (s, 1H, Ar-3″OH), 12.001 (s, 1H, 4″-COOH); ^13^C-NMR (DMSO, 100 MHz, δ ppm): 171.2 (CO-NH), 170.1 (COOH), 163.4 (C-3″), 159.2 (C-4′), 135.4 (C-1″), 135.1 (C-1′), 130.7 (C-5″), 126.8 (C-2′, C-6′), 116.2 (C-6″), 114.0 (C-3′, C-5′), 110.1 (C-4″), 100.4 (C-2″), 64.8 (C-2), 54.8 (C-4), 52.4 (4-OCH3), 31.6 (C-5); Anal. Calcd. for C_18_H_18_N_2_O_5_S: C, 60.02; H, 5.11; N, 8.24; O, 20.98; S, 9.11. Found: C, 60.29; H, 5.22; N, 8.38; O, 21.40; S, 8.16.

#### 3.3.14. *N*,2-Bis(4-methoxyphenyl)-1,3-thiazolidine-4-carboxamide (**4e**)

Brown solid; yield: 66%; m.p.: 129–131 °C; IR cm^−1^: 3150 (N-H stretch), 1600 (C=O), 1500 (N-H bend), 1410 (C=C); ^1^H-NMR (DMSO, δ ppm): 3.445 (d, J = 14.33, 1H, 5-H_a_), 3.545 (d, J = 14.33, 1H, 5-H_b_), 3.742 (s, 3H, Ar-2′OCH_3_), 3.763 (s, 3H, Ar-4″OCH_3_), 3.825 (dd, ^1^J = 9.52, ^2^J = 5.39, 1H, 4-H), 5.142 (s, 1H, 2-H), 6.637–7.297 (m, 8H, Ar-H), 8.512 (s, 1H, amide-NH); ^13^C-NMR (DMSO, 100 MHz, δ ppm): 171.5 (CO-NH), 159.8 (C-4′), 138.1 (C-1″), 135.0 (C-1′), 127.7 (C-2′, C-6′), 121.5 (C-2″, C-6″), 115.5 (C-3″, C-5″), 114.1 (C-3′, C-5′), 64.2 (C-2), 56.5 (C-4), 56.0(4′-OCH3, 4″-OCH3), 32.6 (C-5); Anal. Calcd. for C_18_H_20_N_2_O_3_S: C, 61.94; H, 4.95; N, 7.89; O, 13.94; S, 8.85. Found: C, 61.85; H, 4.50; N, 7.42; O, 13.78; S, 9.22.

### 3.4. Biological Evaluation

#### 3.4.1. Determination of Antioxidant Activity

The synthesized compounds were evaluated for their antioxidant activity by measuring their ability to scavenge DPPH free radicals. A previously described protocol was followed for this purpose [27]. An amount of 20 µL of each of the four different dilutions of test samples (200, 66.66, 22.22, and 7.41 µg/mL) was added to 96-well plates containing 180 µL of DPPH solution. The absorbance at 517 nm was determined after incubating for 30 min at 37 °C. Ascorbic acid (1 mg/mL DMSO) was used as the positive control, and the assay was performed in triplicate. The IC_50_ values were also calculated using the % of inhibitions at different concentrations using Table Curve 2D Windows version 4.07.

#### 3.4.2. Inhibitory Assay against Tyrosinase Enzyme

A tyrosinase inhibitory assay was conducted with the mushroom tyrosinase enzyme [29,30,31]. An amount of 20 µL of mushroom tyrosinase (30 U/mL) was combined with 140 µL (20 mM, pH 6.8) of phosphate buffer. An amount of 20 µL of inhibitor solution was added to a 96-well microplate. Then pre-incubation at room temperature was conducted for 10 min. A solution of l-DOPA (20 μL—0.85 mM) was added. This was followed by the incubation of the plate for 20 min at 25 °C. Using a microplate reader (OPTIMax, Tunable), the absorbance of dopa-chrome was recorded at 492 nm. Kojic acid and phosphate buffer served as the reference inhibitor and negative control, respectively.

The absorbance of dopa-chrome was measured at 492 nm using a microplate reader (OPTIMax, Tunable, San Jose, CA, USA). Phosphate buffer and kojic acid were used as the negative control and the reference inhibitor, respectively. The IC_50_ values were used to express the extent of inhibition. The experiments were performed in triplicate for each concentration.

### 3.5. In Silico Studies

Chemo-informatic analysis was performed using Molinspiration and ChemSketch [32]. Molecular docking was conducted using PyRX v0.8 [33]. A tyrosinase enzyme of mushroom origin having the PDB ID: 2Y9X was acquired from the RCSB Protein DataBank Site [7]. The search space for molecular docking was adjusted to the center with X: −7.4, Y: −23.5, and Z: −32.5 and dimensions of X: 61.33, Y: 57.4, and Z: 63. Discovery studio was used for the visualization of ‘.pdb’ files, cleaning of the protein molecule, and visualization of ligand–protein interactions in 3D and 2D. The structures of the ligands were drawn using ChemDraw v16 and converted into pdb files using Chem3D v16 [34].

## 4. Conclusions

This study presents the design and synthesis of a series of thiazolidine derivatives. The synthesized compounds were screened for their potential biological activity using molecular docking and in vitro antioxidant and tyrosinase inhibitory assays. were conducted to identify the potential biological activity of the synthesized compounds. Compound **2d** showed the maximum antioxidant potential (18.17 ± 1.0 µg/mL). In the tyrosine inhibition assay, compound **3c** exhibited significant activity with an IC_50_ value of 16.5 ± 0.37 µM. Molecular docking analysis led to an overview of the structural properties that can affect ligand–protein interactions. Hydrophobic interactions and hydrogen bonding were found to play a significant role. Compounds **4e**, **3e**, **2d,** and **2a** can also serve as leads for designing tyrosinase inhibitors by further optimizing their structures. These inhibitors have the potential to be used for treating tyrosinase-related disorders in various fields, including medicine, food, agriculture, and cosmetics. Future prospects may include in vivo analyses, such as skin depigmentation assay, and mechanism-based studies for evaluating the effects of the synthesized compounds in biological systems. Being novel in nature, these compounds can also be screened for other biological activities.

## Figures and Tables

**Table 1:** Physical properties of synthesized derivatives.

Compound	Physical State	Color	Molecular Formula	Melting Point (°C)	%Yield
**1a**	Solid	Yellow	C_17_H_16_N_2_O_4_S	135–136	54
**1b**	Solid	Orange	C_14_H_18_N_2_O_3_S	128–129	68
**1c**	Solid	Black	C_16_H_16_N_2_O_3_S	109–111	45
**1d**	Solid	Yellow	C_17_H_16_N_2_O_5_S	134–135	68
**1e**	Solid	Yellow	C_17_H_18_N_2_O_3_S	83–84	60
**2a**	Solid	White	C_18_H_18_N_2_O_4_S	116–117	20
**2d**	Solid	White	C_18_H_18_N_2_O_5_S	112–113	25
**3a**	Solid	Yellow	C_17_H_16_N_2_O_4_S	164–166	72
**3c**	Solid	Black	C_16_H_16_N_2_O_3_S	174–176	68
**3d**	Solid	Yellow	C_17_H_16_N_2_O_5_S	130–131	68
**3e**	Solid	Brown	C_17_H_18_N_2_O_3_S	115–117	65
**4a**	Solid	Yellow	C_18_H_18_N_2_O_4_S	94–95	60
**4d**	Solid	White	C_18_H_18_N_2_O_5_S	109–110	66
**4e**	Solid	Brown	C_18_H_20_N_2_O_3_S	129–131	66

**Table 2:** Inhibitory activity of synthesized derivatives against the tyrosinase enzyme.

Compound	IC_50_ ± SEM (µM)
**1a**	98.5 ± 10.6
**1b**	131.5 ± 20.3
**1c**	ND
**1d**	256.4 ± 16.3
**1e**	109.3 ± 13.4
**2a**	49.8 ± 11.9
**2d**	46.6 ± 7.6
**3a**	186.3 ± 18.3
**3c**	16.5 ± 0.37
**3d**	ND
**3e**	44.8 ± 6.2
**4a**	ND
**4d**	ND
**4e**	41.6 ± 10.4
Kojic acid	15.9 ± 2.5

ND: Not determined.

**Table 3:** Binding affinities and respective amino acids interacting with the ligand (synthesized derivatives).

Protein Target	Ligand	Binding Affinity (Kcal/mol) ^†^	Amino Acids Involved
Mushroom Tyrosinase PDB ID: 2Y9X	**1a**	−7.3	HIS61, HIS259, PRO277, SER282, VAL283
	**1b**	−7.4	HIS263, PRO277, MET280, SER282, VAL283, ALA286
	**1c**	−8.3	VAL248, ASN260, HIS263, PHE264, VAL283, ALA286
	**1d**	−7.5	HIS61, HIS259, HIS263, PRO277, MET280, SER282, VAL283
	**1e**	−6.9	PRO277, HIS259, HIS263, VAL283
	**2a**	−7.3	HIS61, HIS259, PRO277, SER282, VAL282
	**2d**	−7.5	HIS61, HIS259, HIS263, PRO277, SER282, VAL283
	**3a**	−7.4	HIS244, VAL248, VAL283
	**3c**	−8.4	VAL248, ASN260, HIS263, PHE264, VAL283, ALA286
	**3d**	−7.1	HIS61, HIS259, ASN260, HIS263, PRO277, SER282, VAL283
	**3e**	−6.9	HIS244, VAL248, HIS263, VAL283, GLU322
	**4a**	−7.4	HIS244, VAL248, HIS263, MET280, SER282, VAL283, GLU322
	**4d**	−7	HIS61, HIS259, HIS263, PRO277, MET280, SER282, VAL283
	**4e**	−7.1	HIS244, GLY245, VAL248, HIS263, VAL283
	Kojic acid	−5.6	HIS263, VAL283, ALA286

^†^ All values are expressed as the mean of triplicate determinations.

## Data Availability

Data are contained within the article and the Supplementary Materials.

## Supplementary Materials

The following supporting information can be downloaded at: https://www.mdpi.com/article/10.3390/ph16060835/s1; Table S1: Chemo-informatic analysis of synthesized derivatives; Figures S1–S8: NMR and FTIR spectra of selected compounds; Figures S9–23: Binding interactions between derivatives and mushroom tyrosinase active site.
