# Supplementary material for: Synthesis, In Silico Studies, and Antioxidant and Tyrosinase Inhibitory Potential of 2-(Substituted Phenyl) Thiazolidine-4-Carboxamide Derivatives
Source: Pharmaceuticals (Basel). 2023 Jun 2;16(6):835. doi: 10.3390/ph16060835 (PMC10303221; doi:10.3390/ph16060835)
Supplement: Supplementary file 1 [file pharmaceuticals-16-00835-s001.zip › pharmaceuticals-2213020-supplementary.pdf]

Table S1. Chemo-informatic analysis of synthesized derivatives

Compound	No. of HBA	No. of HBD	Mol. Log*P*	Polar surface area (PSA) (Å^2)	Density (g/cm^3)	Molar volume (cm^3)	Lipinski rule validation
**1a**	6	4	2.69	98.65	1.447 ± 0.06	237.9 ± 3.0	yes
**1b**	5	2	1.18	61.8	1.321 ± 0.06	222.7 ± 3.0	yes
**1c**	5	4	2.31	81.58	1.413 ± 0.06	223.8 ± 3.0	yes
**1d**	7	5	2.69	118.88	1.524 ± 0.06	236.3 ± 3.0	yes
**1e**	5	3	2.84	70.59	1.324 ± 0.06	249.4 ± 3.0	yes
**2a**	6	3	2.76	87.66	1.360 ± 0.06	263.5 ± 3.0	yes
**2d**	7	4	2.76	107.89	1.429 ± 0.06	261.9 ± 3.0	yes
**3a**	6	4	2.28	98.65	1.447 ± 0.06	237.9 ± 3.0	yes
**3c**	5	4	1.89	81.58	1.413 ± 0.06	223.8 ± 3.0	yes
**3d**	7	5	2.27	118.88	1.524 ± 0.06	236.3 ± 3.0	yes
**3e**	5	3	2.42	70.59	1.324 ± 0.06	249.4 ± 3.0	yes
**4a**	6	3	2.81	87.66	1.360 ± 0.06	263.5 ± 3.0	yes
**4d**	7	4	2.81	107.89	1.429 ± 0.06	261.9 ± 3.0	yes
**4e**	5	2	2.96	59.59	1.252 ± 0.06	275.0 ± 3.0	yes

NMR and FTIR spectra of selected compounds

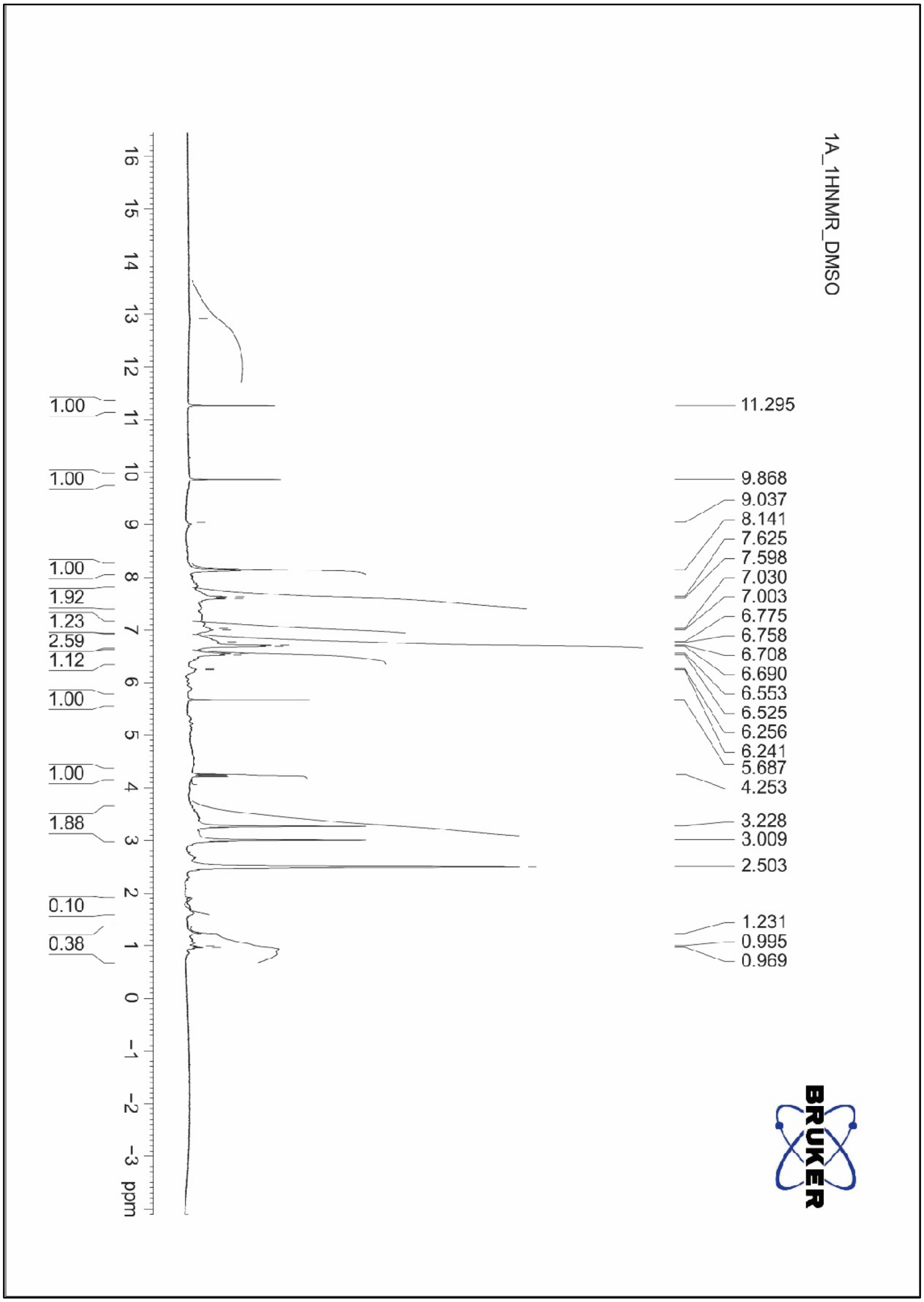

Figure S1. ^{1}H NMR Spectra of Compound 1A

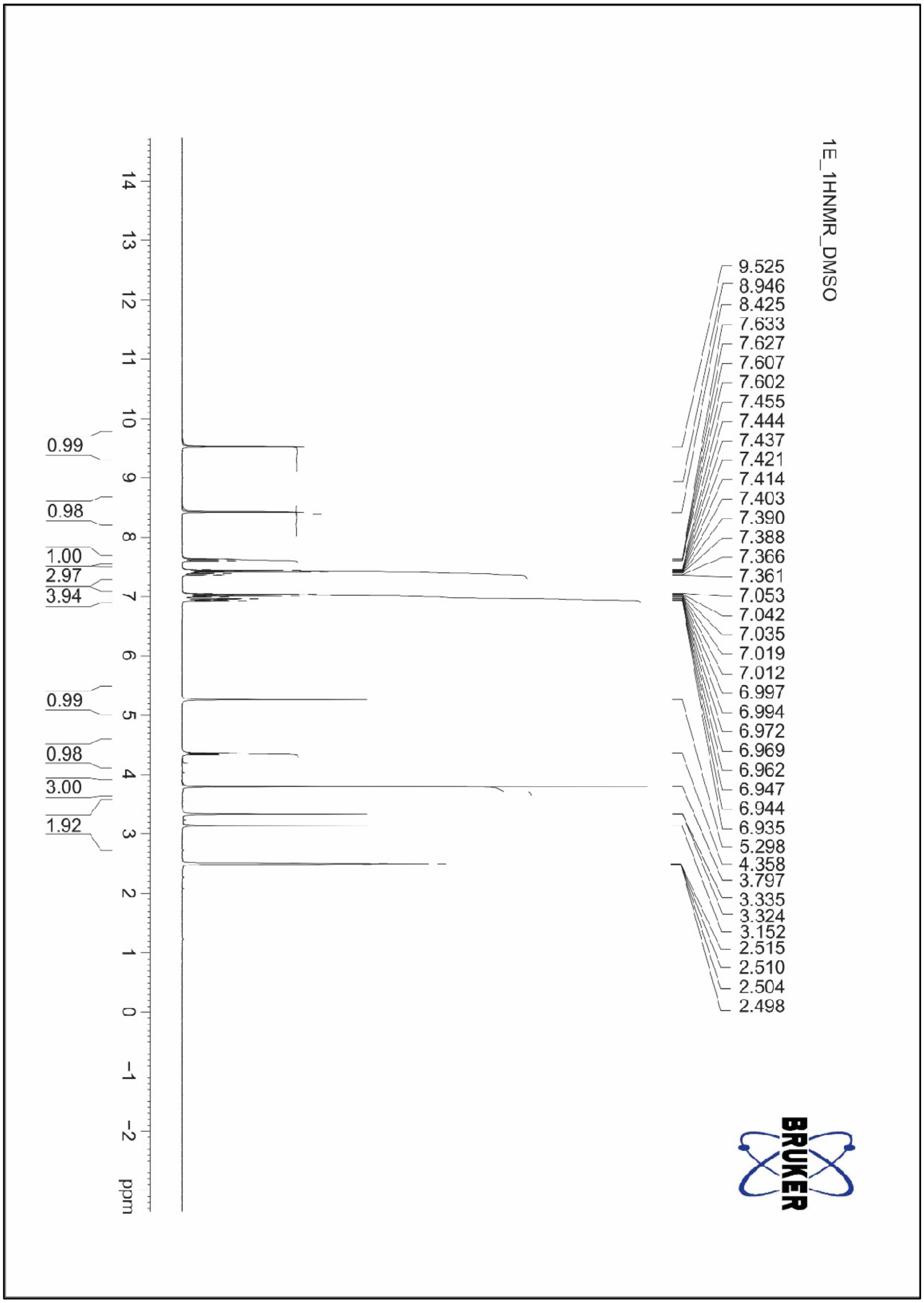

Figure S2. ^{1}H NMR Spectra of Compound 1E

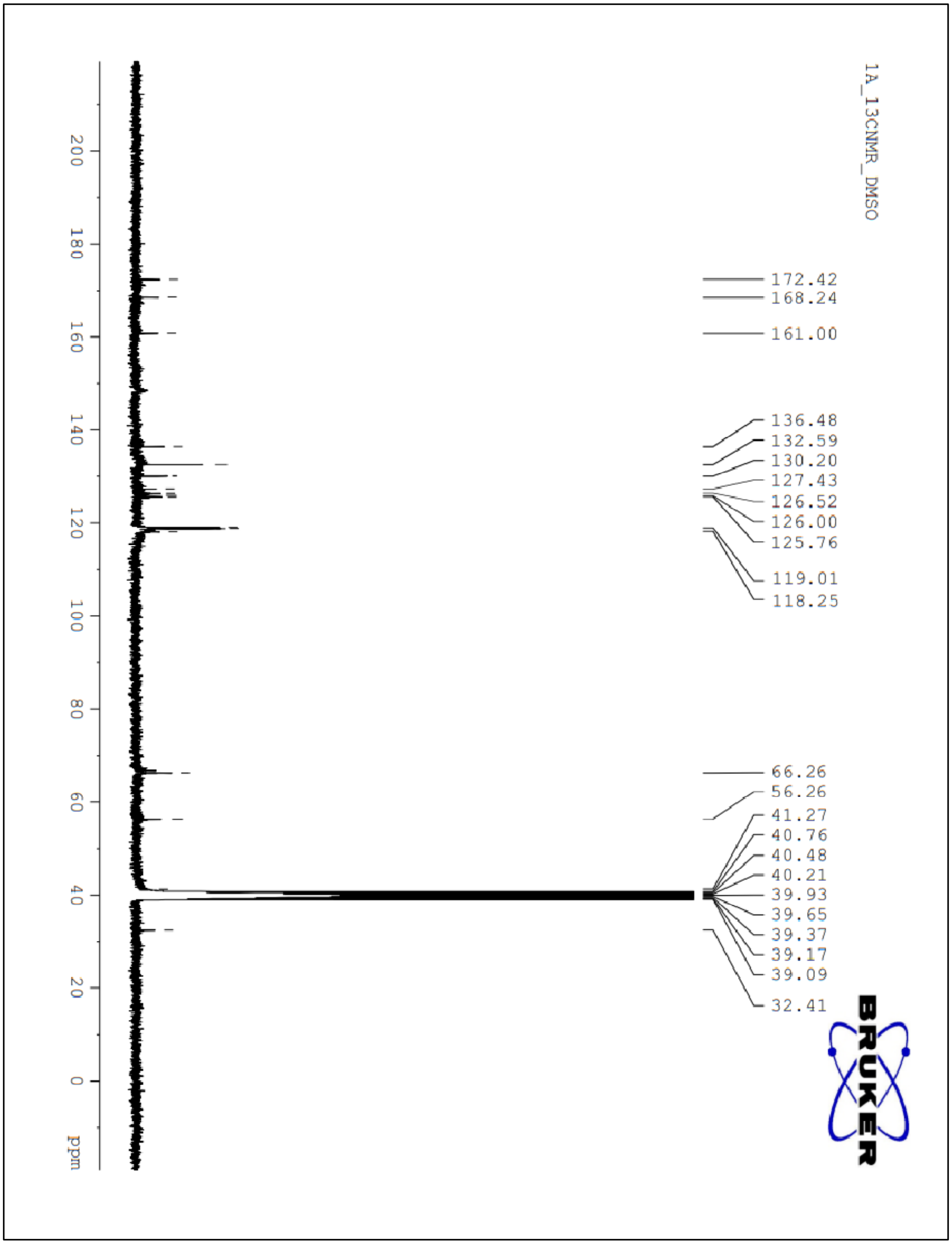

Figure S3. ^{13}C NMR Spectra of Compound 1A

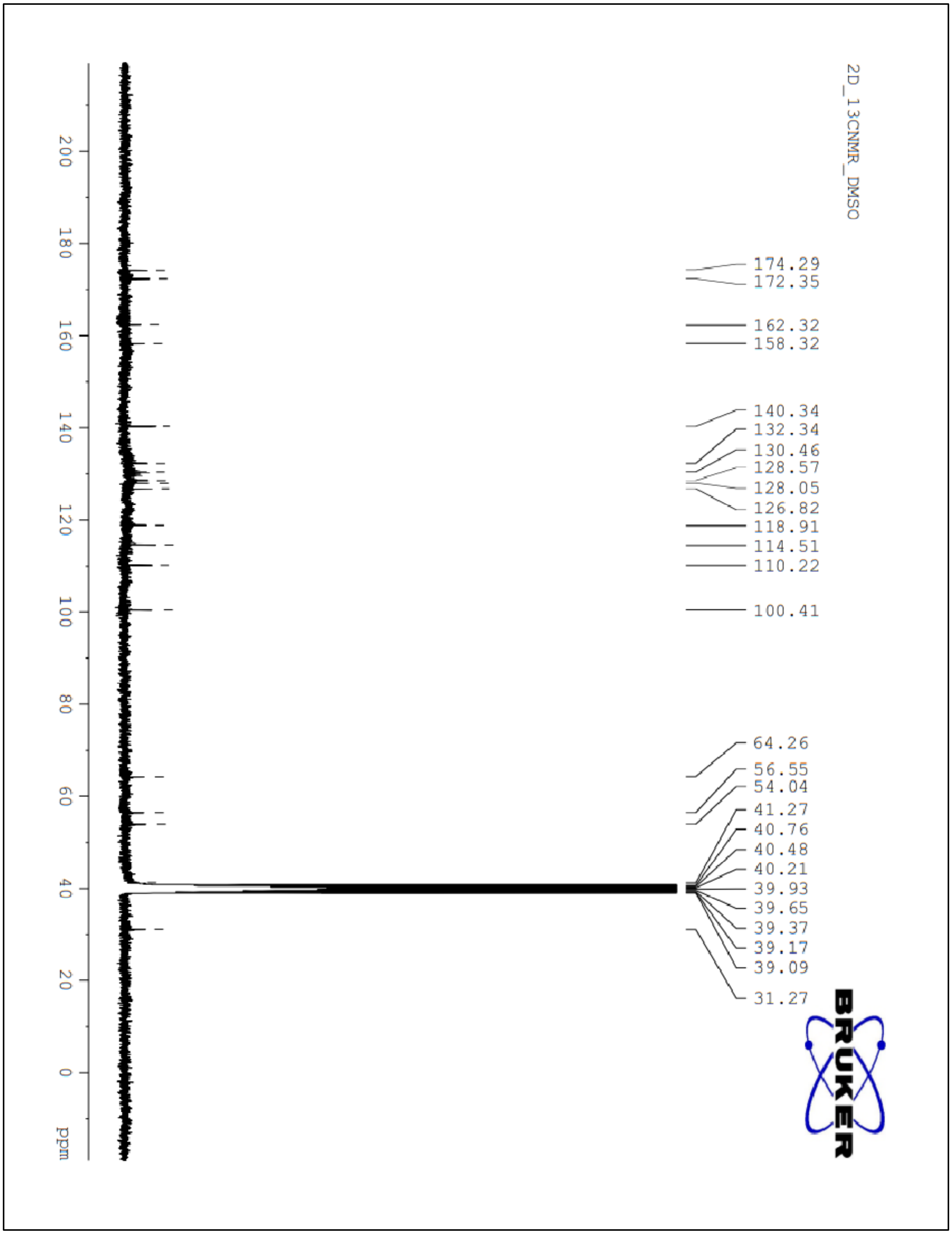

Figure S4. ^{13}C NMR Spectra of Compound 2D

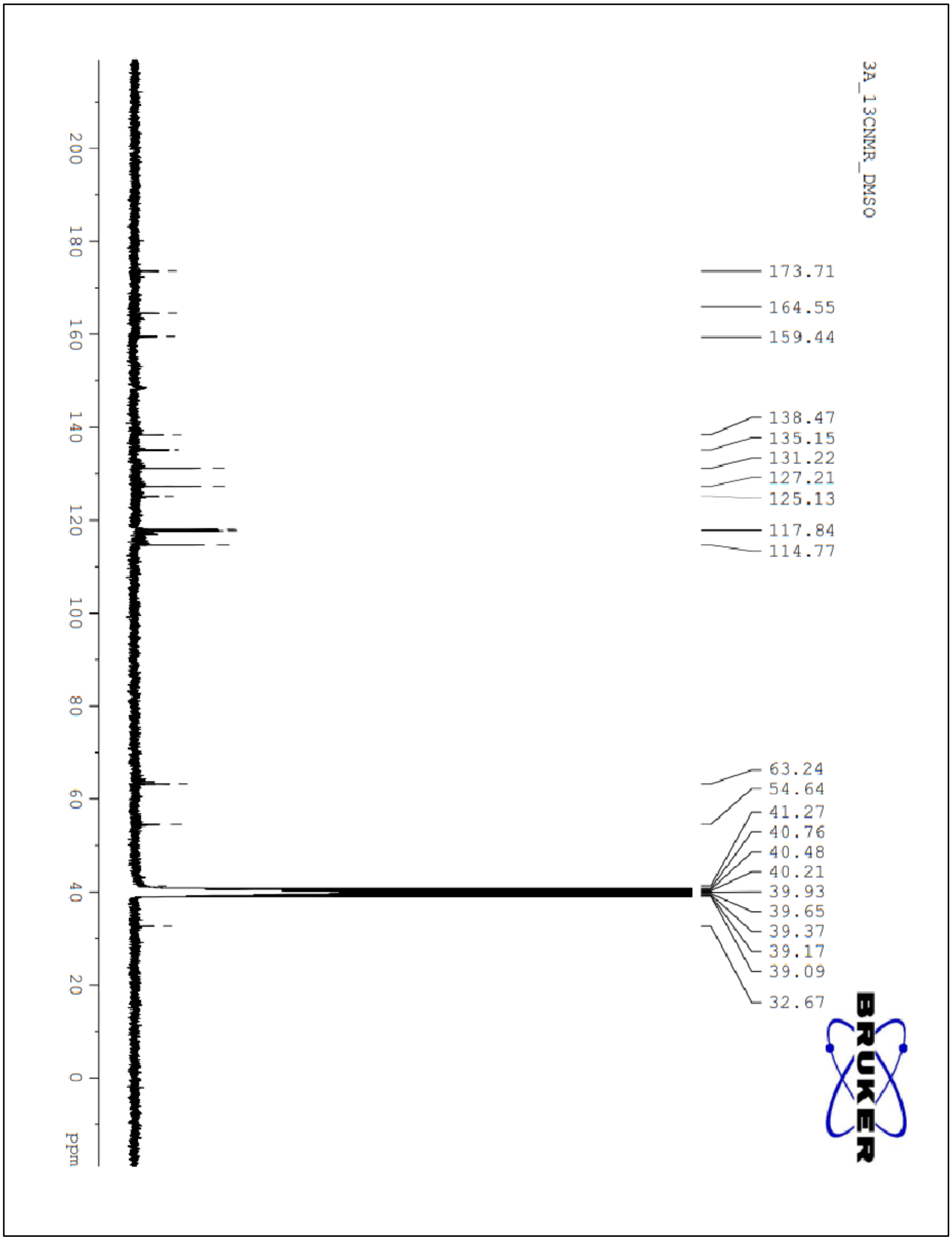

Figure S5. ^{13}C NMR Spectra of Compound 3A

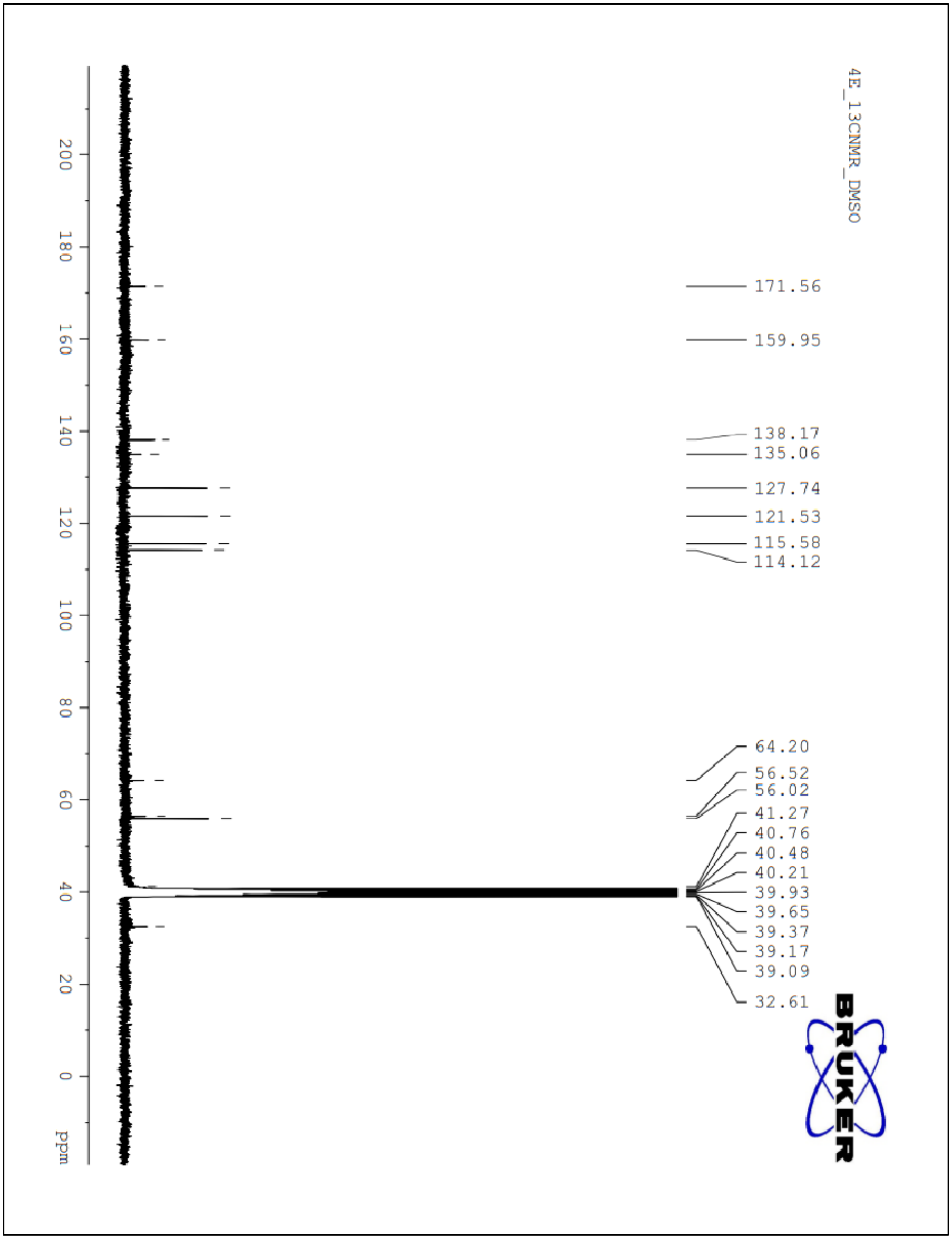

Figure S6. ^{13}C NMR Spectra of Compound 4E

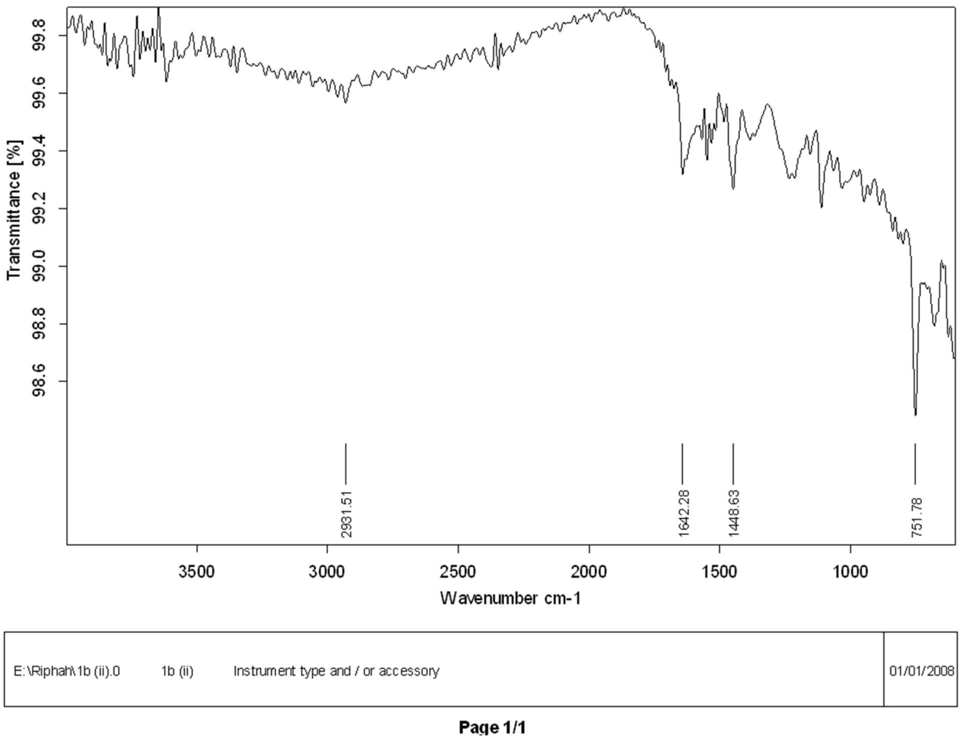

Figure S7. IR spectra of compound 1b

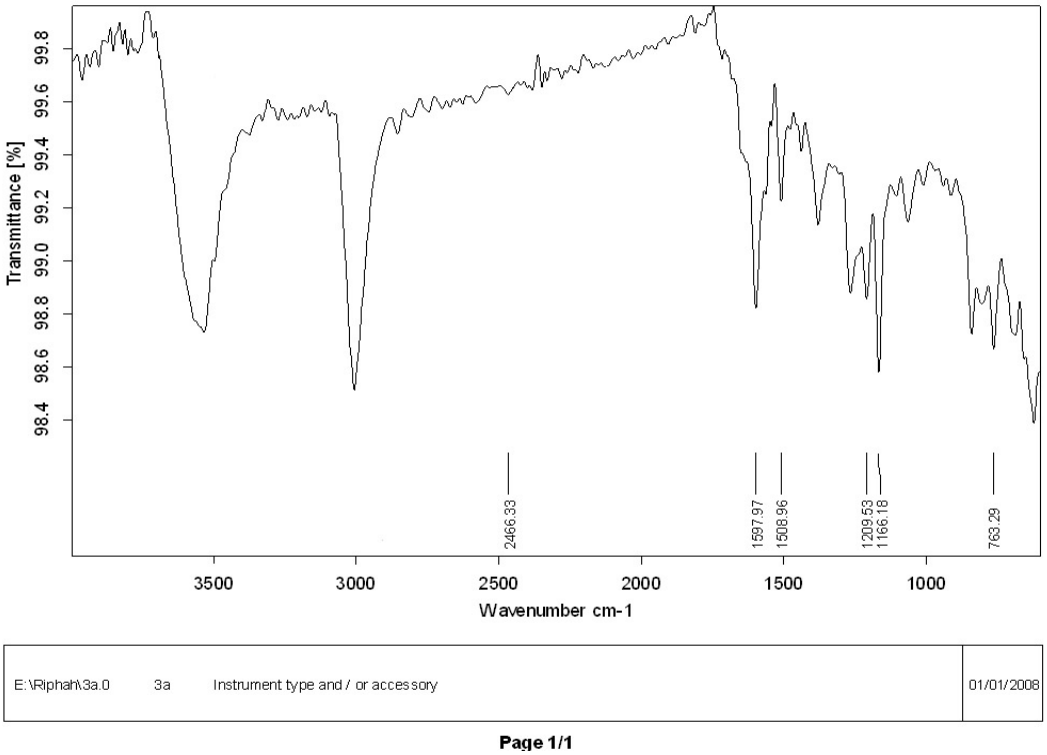

Figure S8. IR spectra of compound 3a

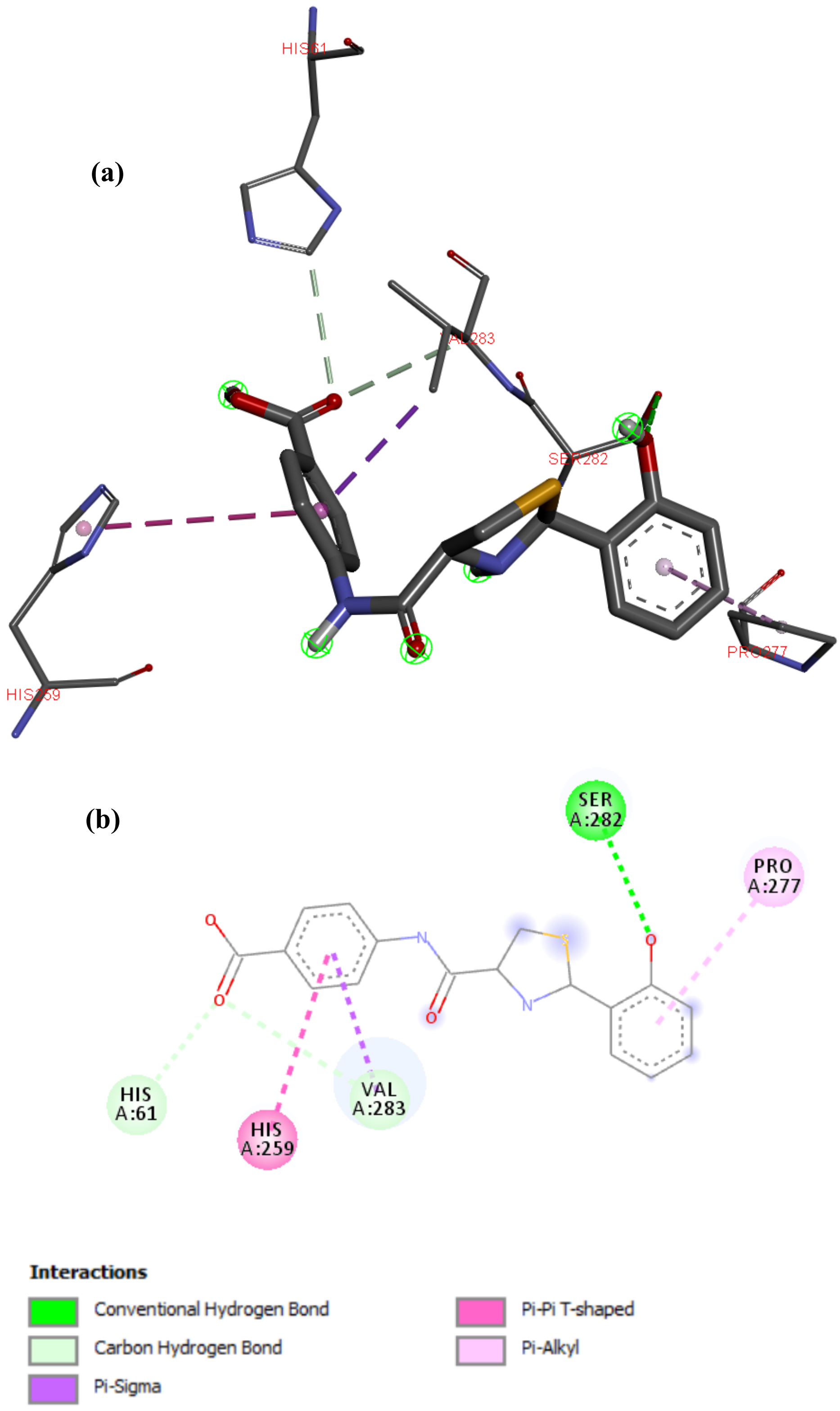

Figure S9. Binding interactions between derivative 1a and mushroom tyrosinase active site (PDB-ID 2Y9X). a) Binding conformation of the compound 1a in three-dimension along with the binding interaction of protein-ligand complex. b) Interaction patterns in 2D indicating the type and distance of the interaction. Legend indicates various types of binding interactions present.

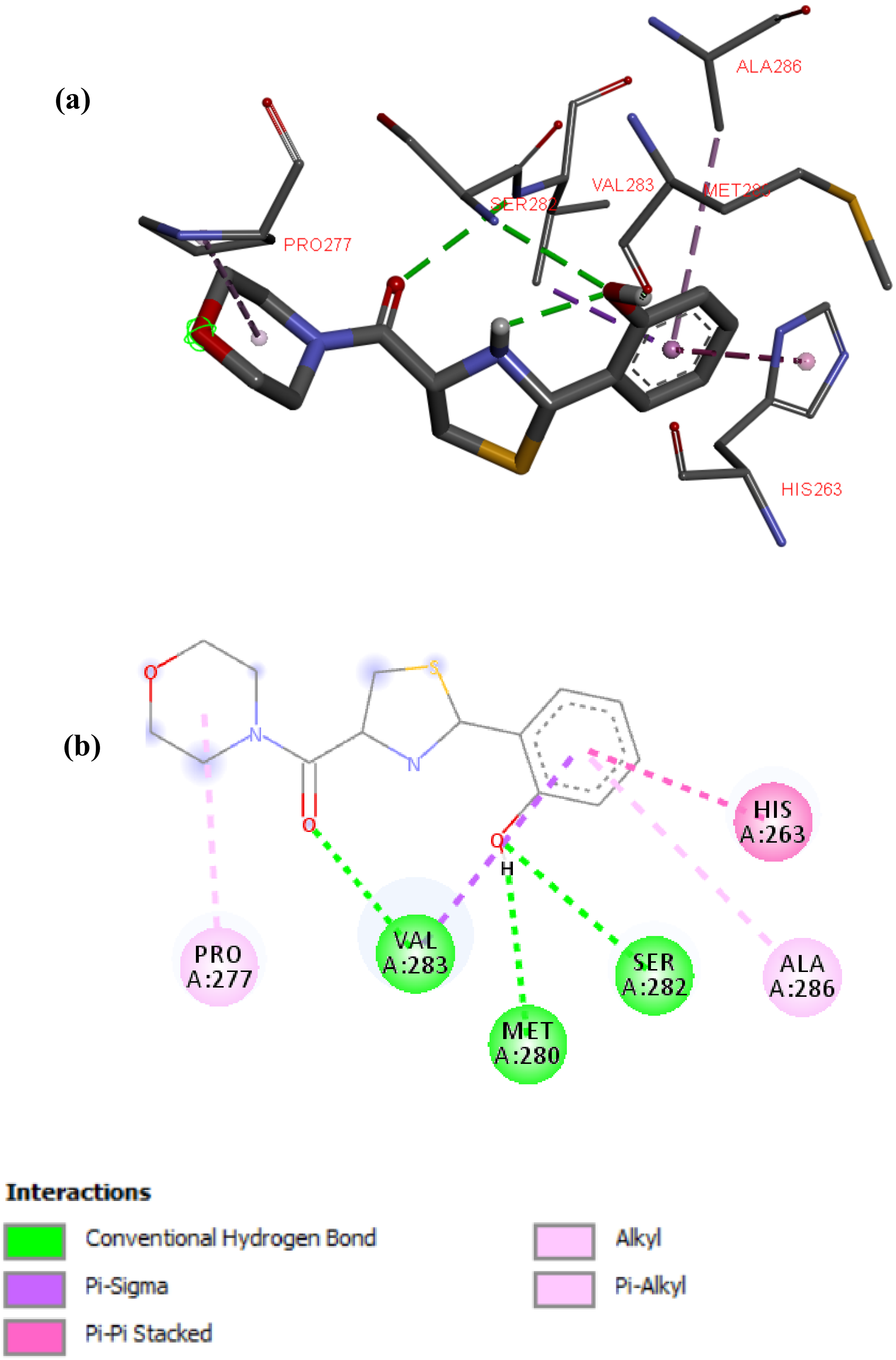

Figure S10. Binding interactions between derivative 1b and mushroom tyrosinase active site (PDB-ID 2Y9X). a) Binding conformation of the compound 1b in three-dimension along with the binding interaction of protein-ligand complex. b) Interaction patterns in 2D indicating the type and distance of the interaction. Legend indicates various types of binding interactions present.

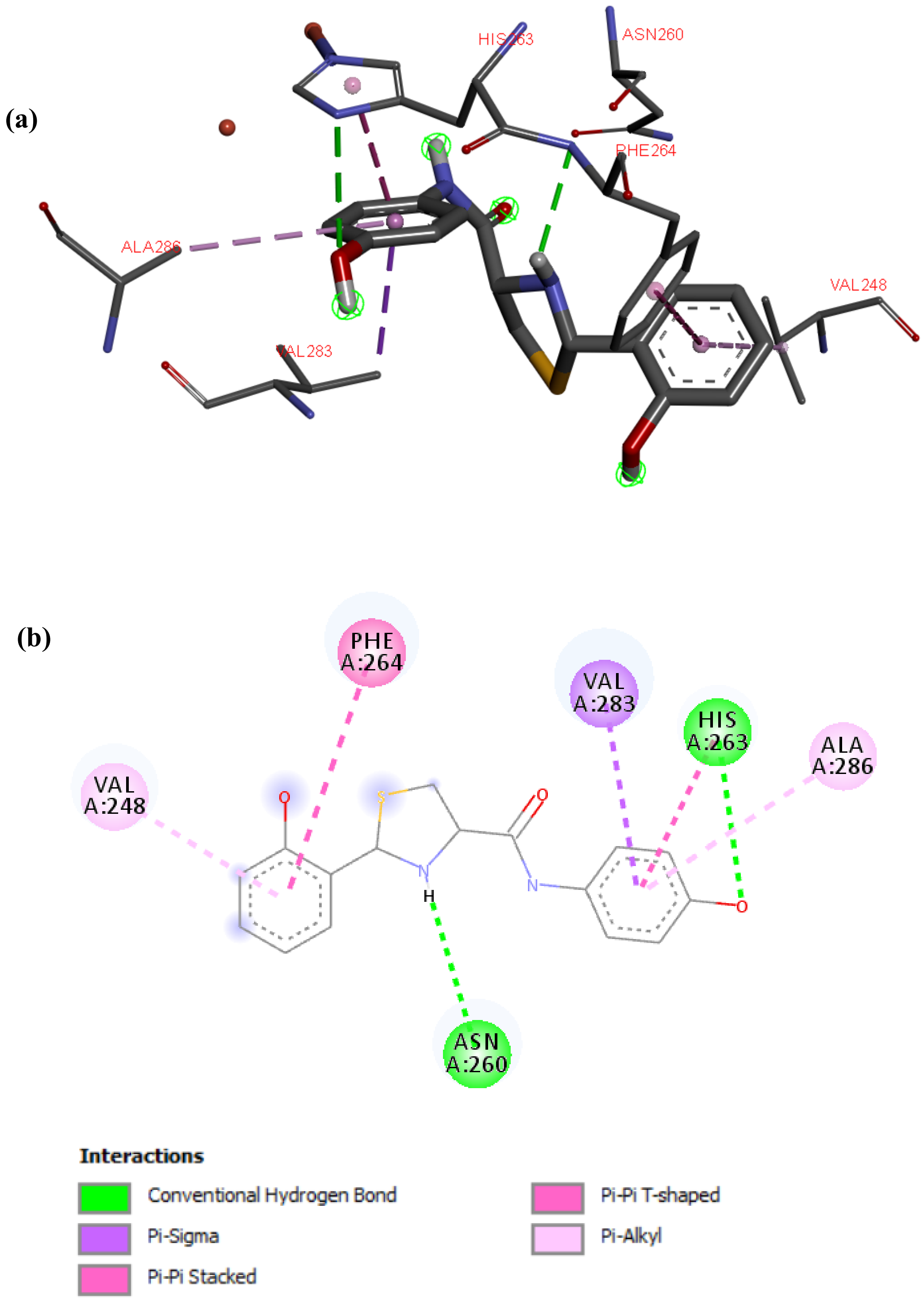

Figure S11. Binding interactions between derivative 1c and mushroom tyrosinase active site (PDB-ID 2Y9X). a) Binding conformation of the compound 1c in three-dimension along with the binding interaction of protein-ligand complex. b) Interaction patterns in 2D indicating the type and distance of the interaction. Legend indicates various types of binding interactions present.

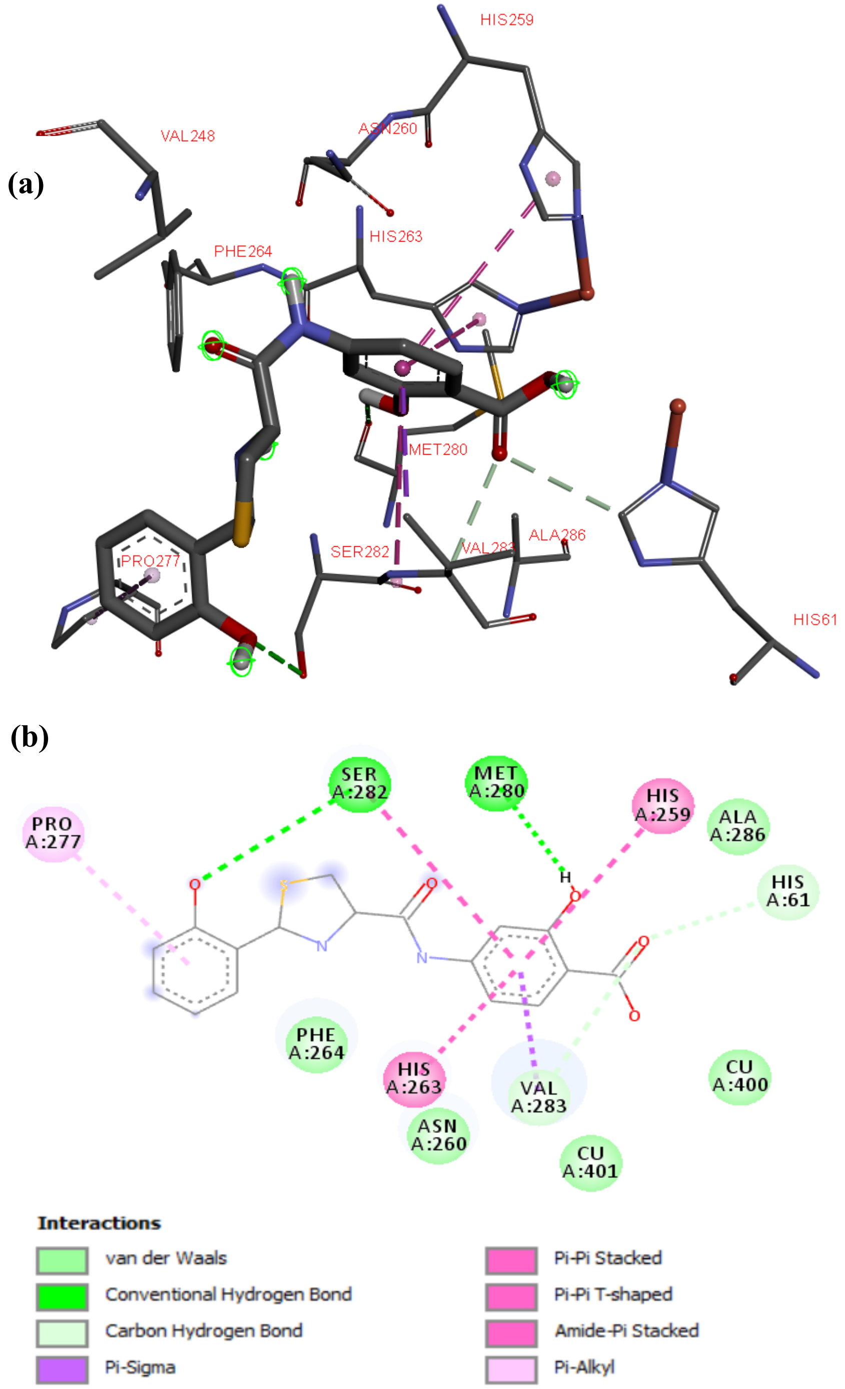

Figure S12. Binding interactions between derivative 1d and mushroom tyrosinase active site (PDB-ID 2Y9X). a) Binding conformation of the compound 1d in three-dimension along with the binding interaction of protein-ligand complex. b) Interaction patterns in 2D indicating the type and distance of the interaction. Legend indicates various types of binding interactions present.

(a)

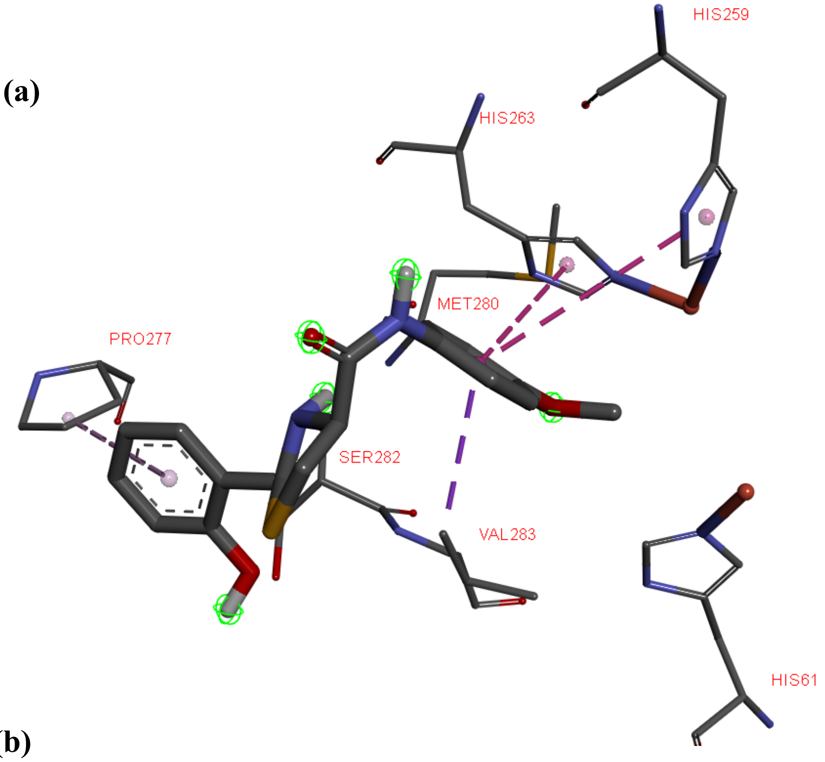

(b)

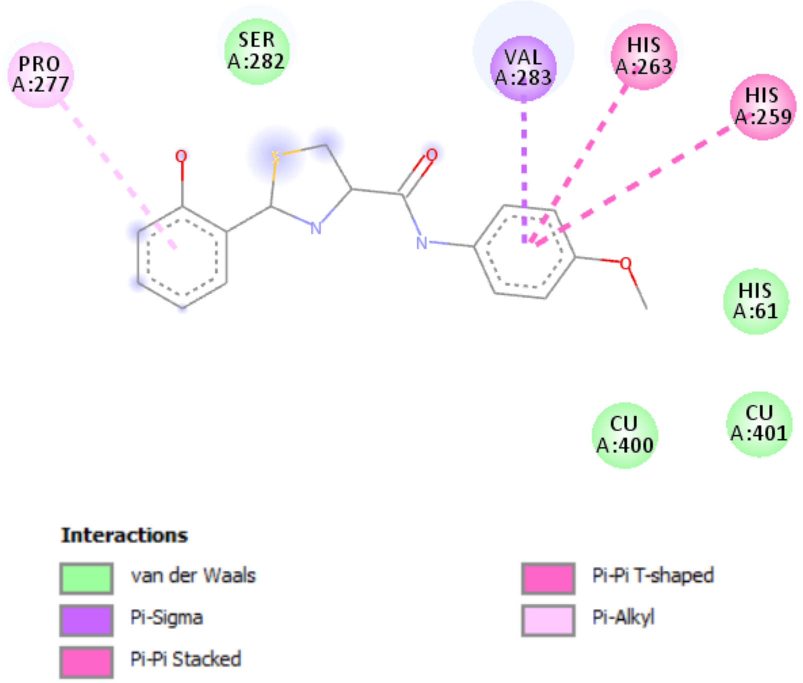

Figure S13. Binding interactions between derivative 1e and mushroom tyrosinase active site (PDB-ID 2Y9X). a) Binding conformation of the compound 1e in three-dimension along with the binding interaction of protein-ligand complex. b) Interaction patterns in 2D indicating the type and distance of the interaction. Legend indicates various types of binding interactions present.

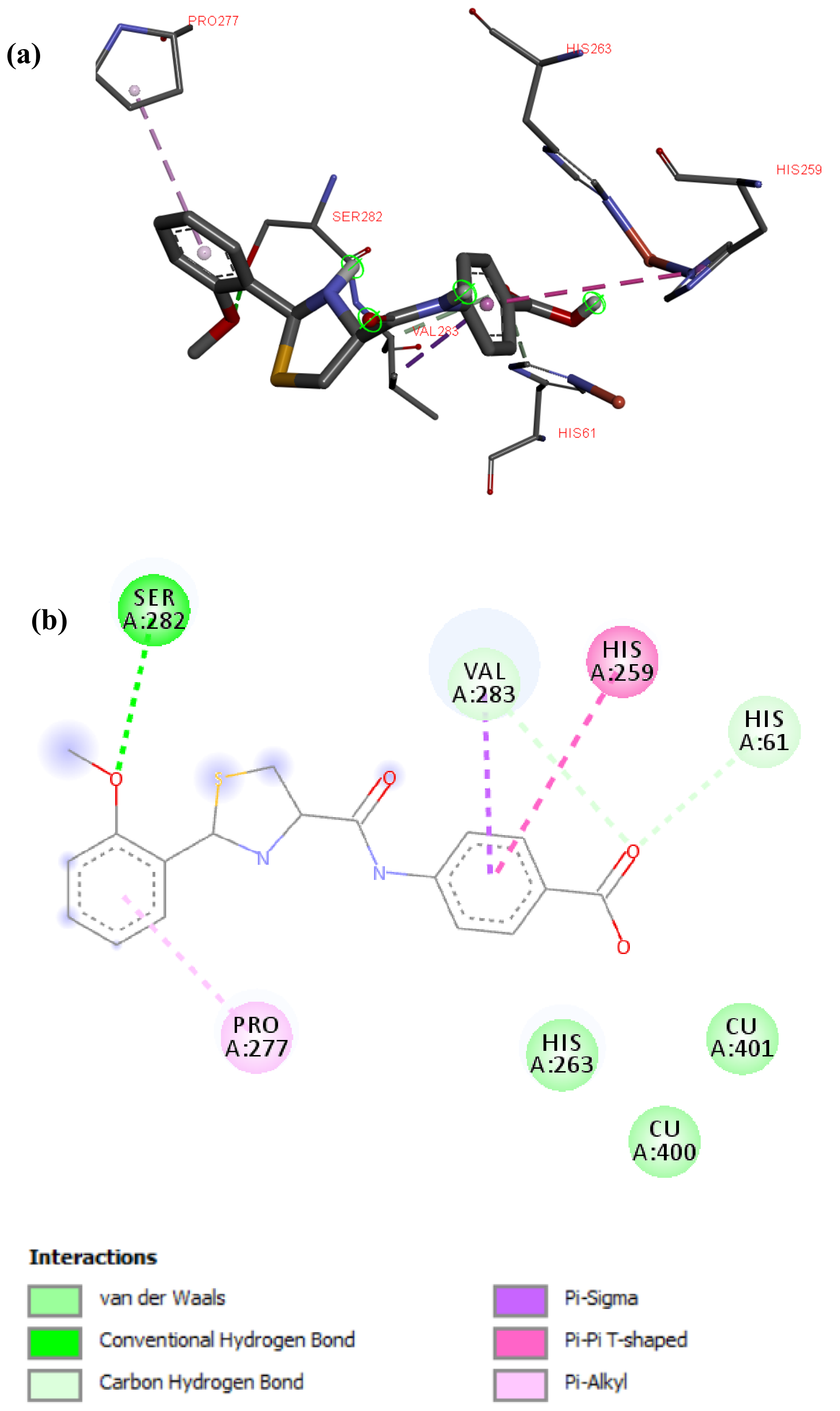

Figure S14. Binding interactions between derivative 2a and mushroom tyrosinase active site (PDB-ID 2Y9X). a) Binding conformation of the compound 2a in three-dimension along with the binding interaction of protein-ligand complex. b) Interaction patterns in 2D indicating the type and distance of the interaction. Legend indicates various types of binding interactions present.

(a)

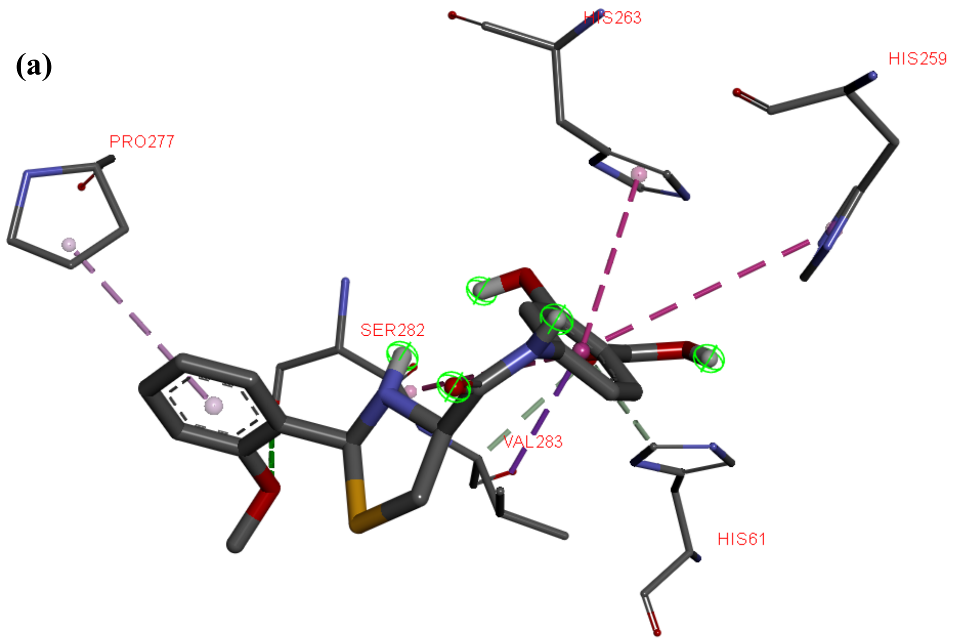

(b)

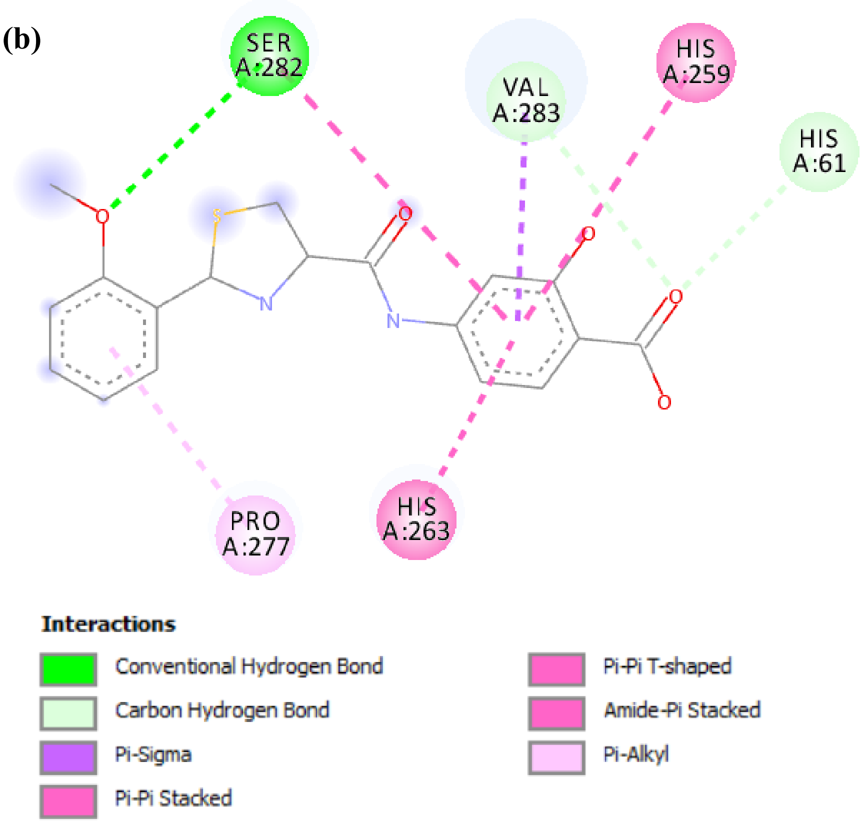

Figure S15. Binding interactions between derivative 2d and mushroom tyrosinase active site (PDB-ID 2Y9X). a) Binding conformation of the compound 2d in three-dimension along with the binding interaction of protein-ligand complex. b) Interaction patterns in 2D indicating the type and distance of the interaction. Legend indicates various types of binding interactions present.

(a)

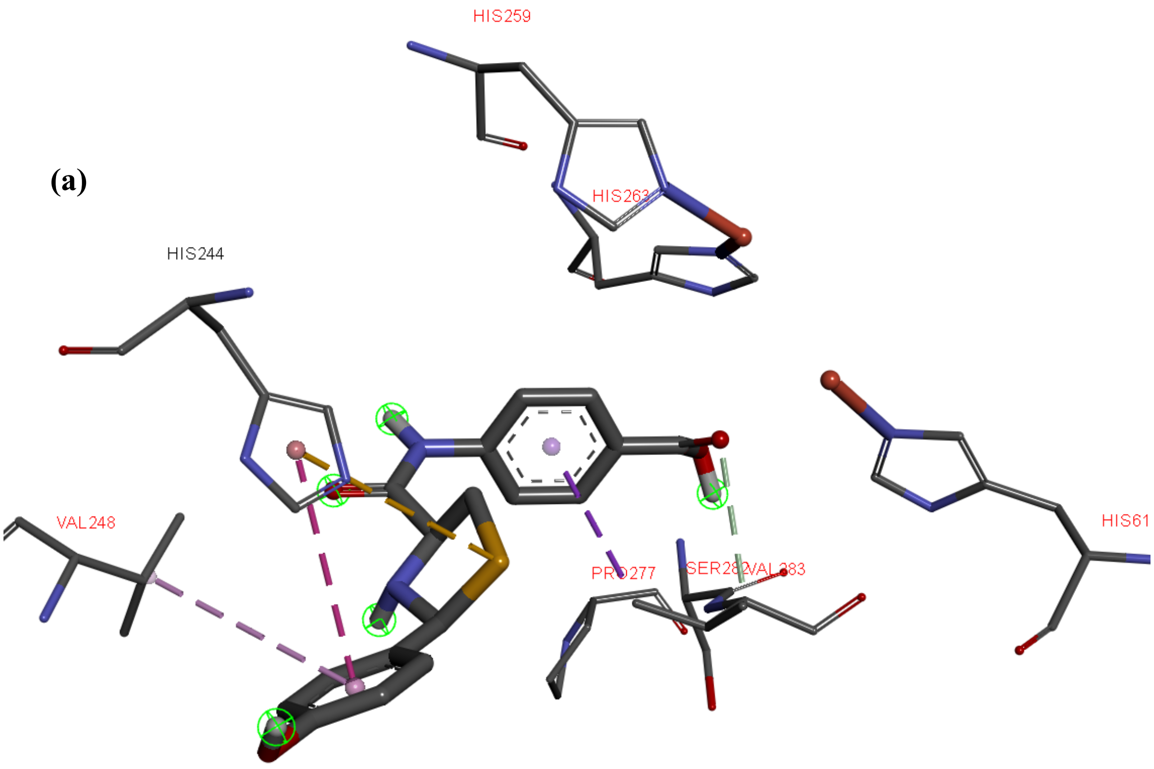

(b)

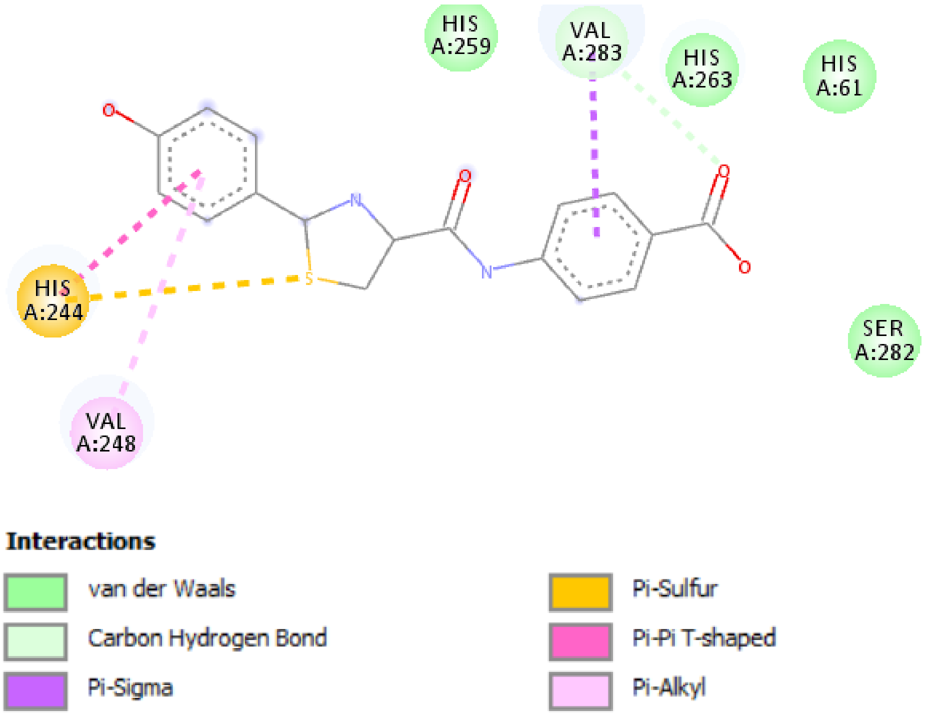

Figure S16. Binding interactions between derivative 3a and mushroom tyrosinase active site (PDB-ID 2Y9X). a) Binding conformation of the compound 3a in three-dimension along with the binding interaction of protein-ligand complex. b) Interaction patterns in 2D indicating the type and distance of the interaction. Legend indicates various types of binding interactions present.

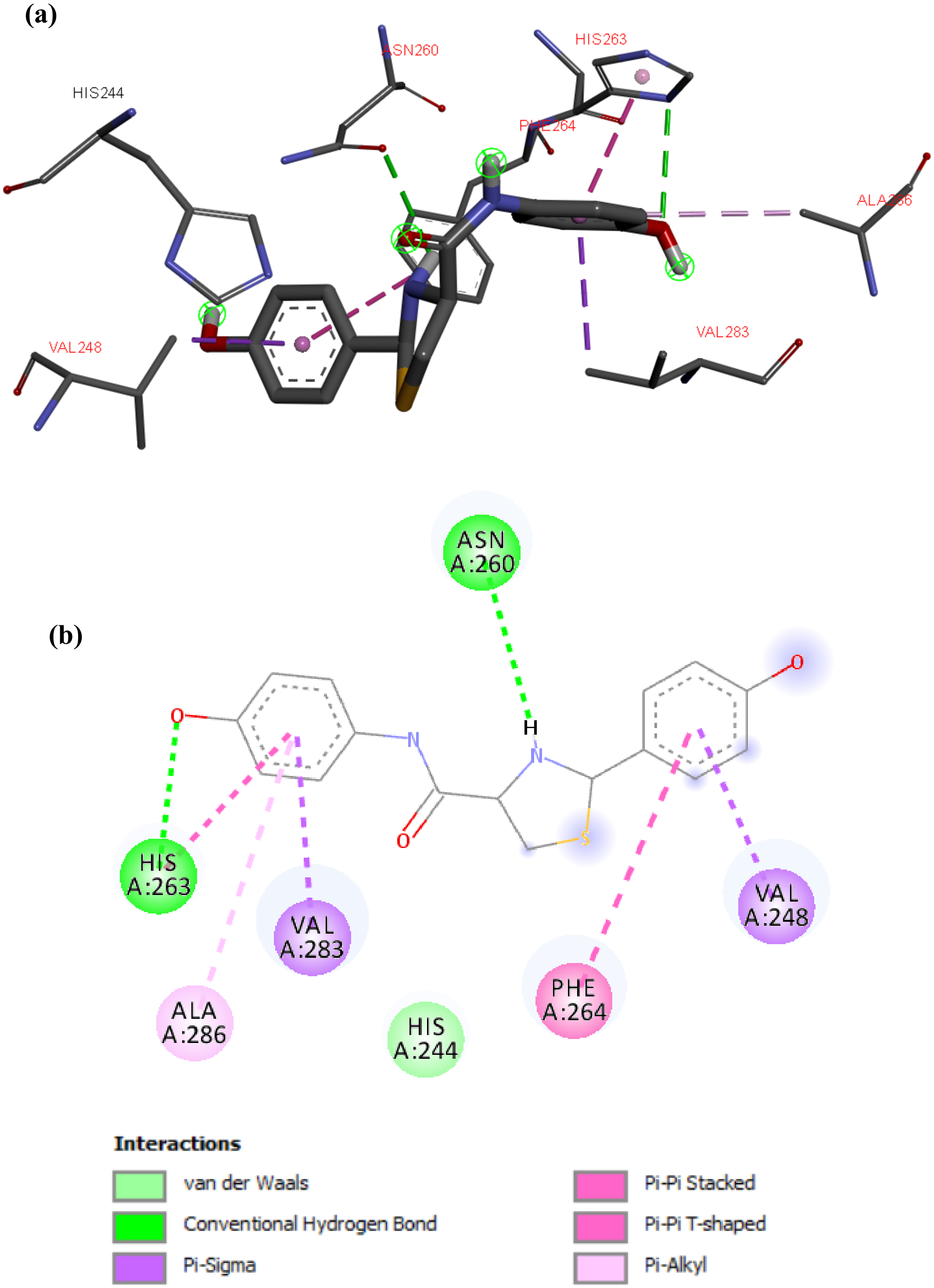

Figure S17. Binding interactions between derivative 3c and mushroom tyrosinase active site (PDB-ID 2Y9X). a) Binding conformation of the compound 3c in three-dimension along with the binding interaction of protein-ligand complex. b) Interaction patterns in 2D indicating the type and distance of the interaction. Legend indicates various types of binding interactions present.

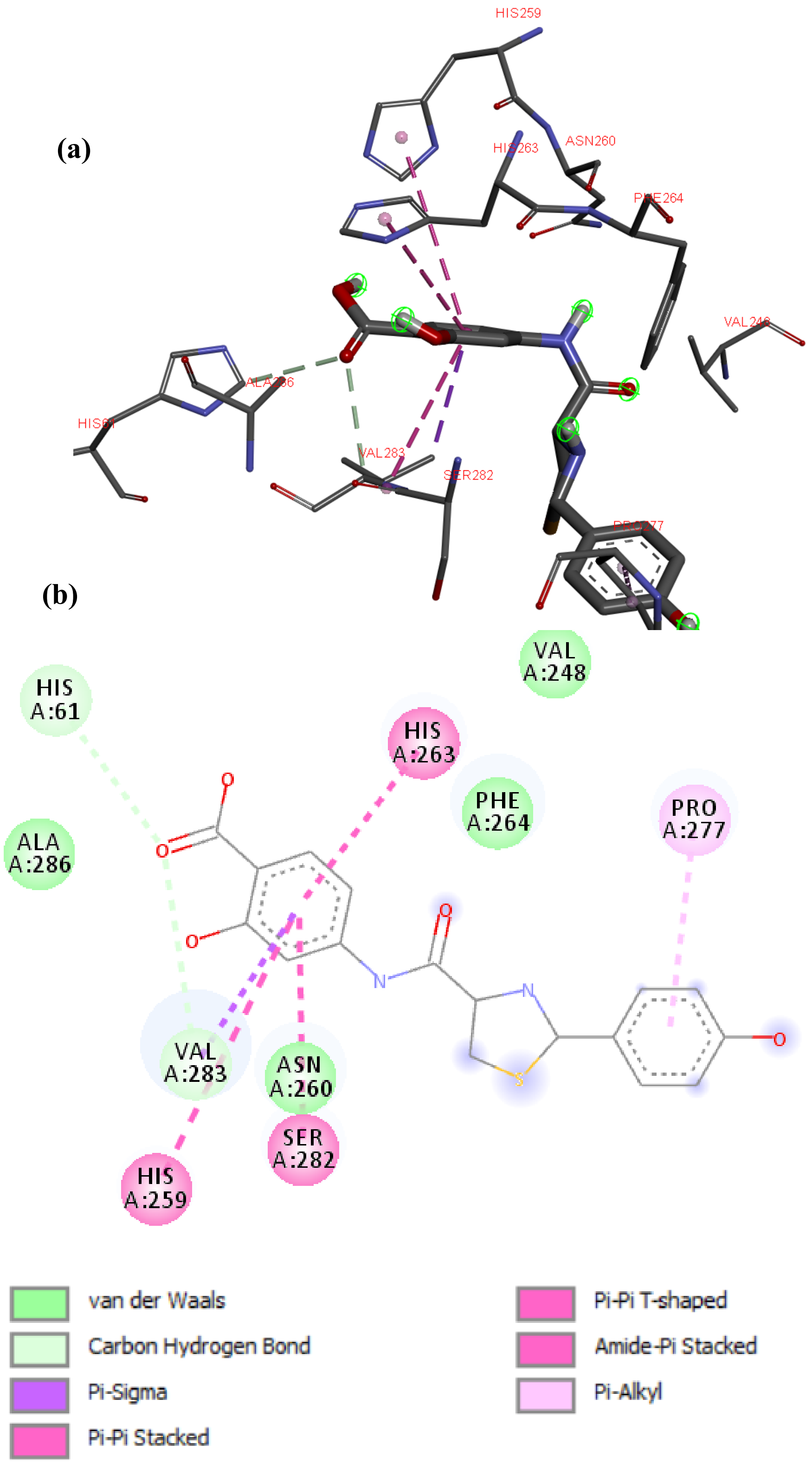

Figure S18. Binding interactions between derivative 3d and mushroom tyrosinase active site (PDB-ID 2Y9X). a) Binding conformation of the compound 3d in three-dimension along with the binding interaction of protein-ligand complex. b) Interaction patterns in 2D indicating the type and distance of the interaction. Legend indicates various types of binding interactions present.

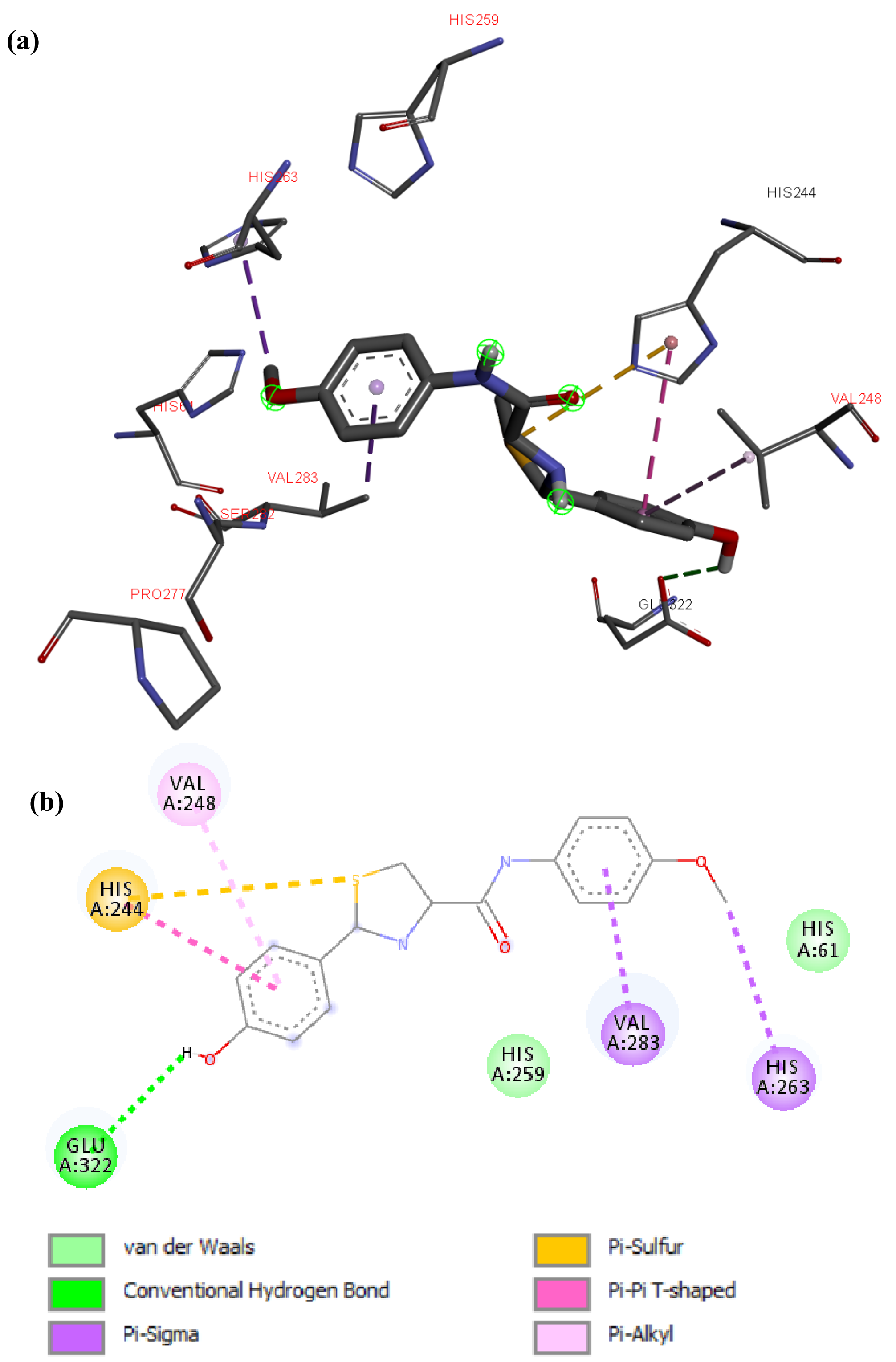

Figure S19. Binding interactions between derivative 3e and mushroom tyrosinase active site (PDB-ID 2Y9X). a) Binding conformation of the compound 3e in three-dimension along with the binding interaction of protein-ligand complex. b) Interaction patterns in 2D indicating the type and distance of the interaction. Legend indicates various types of binding interactions present.

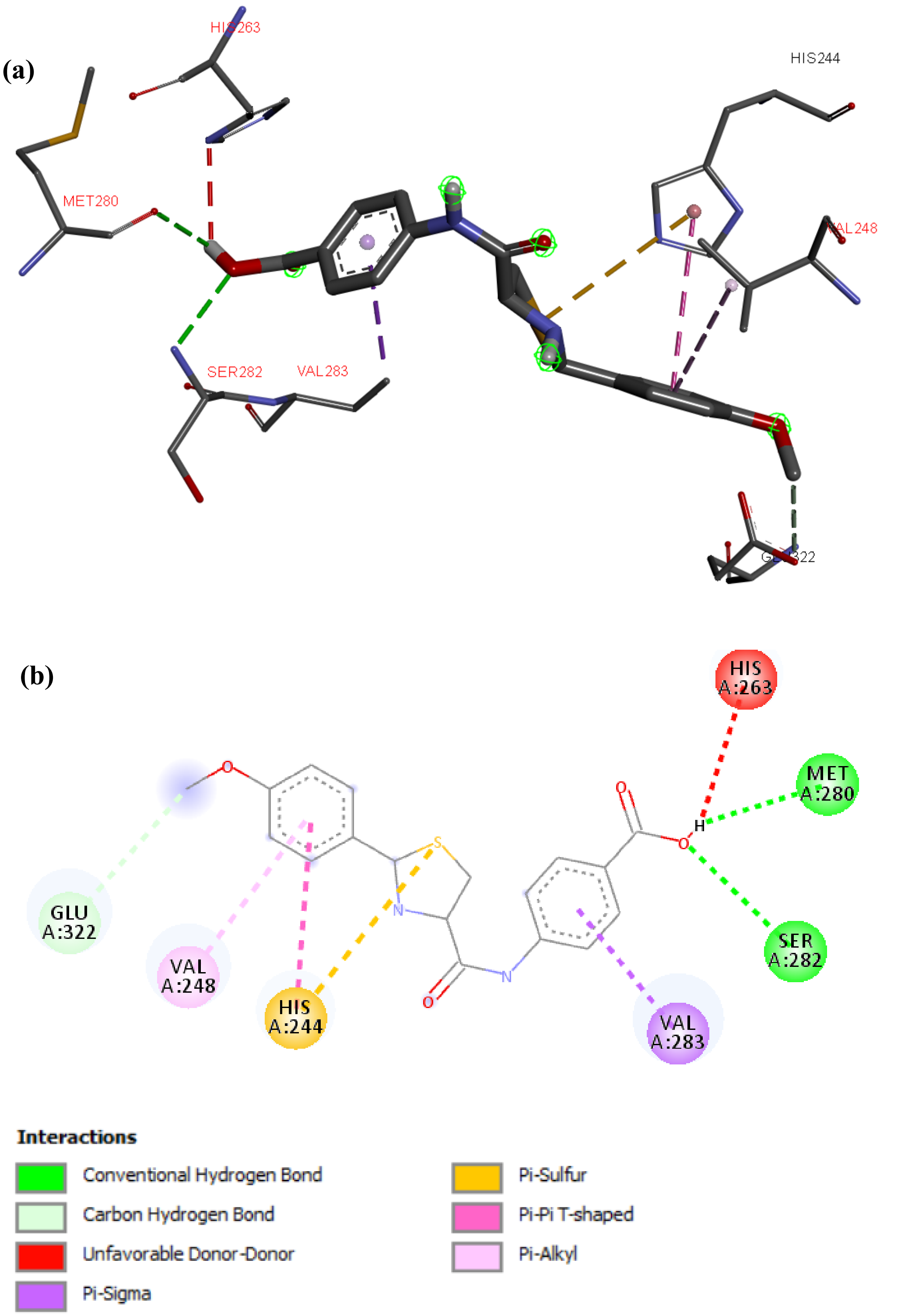

Figure S20. Binding interactions between derivative 4a and mushroom tyrosinase active site (PDB-ID 2Y9X). a) Binding conformation of the compound 4a in three-dimension along with the binding interaction of protein-ligand complex. b) Interaction patterns in 2D indicating the type and distance of the interaction. Legend indicates various types of binding interactions present.

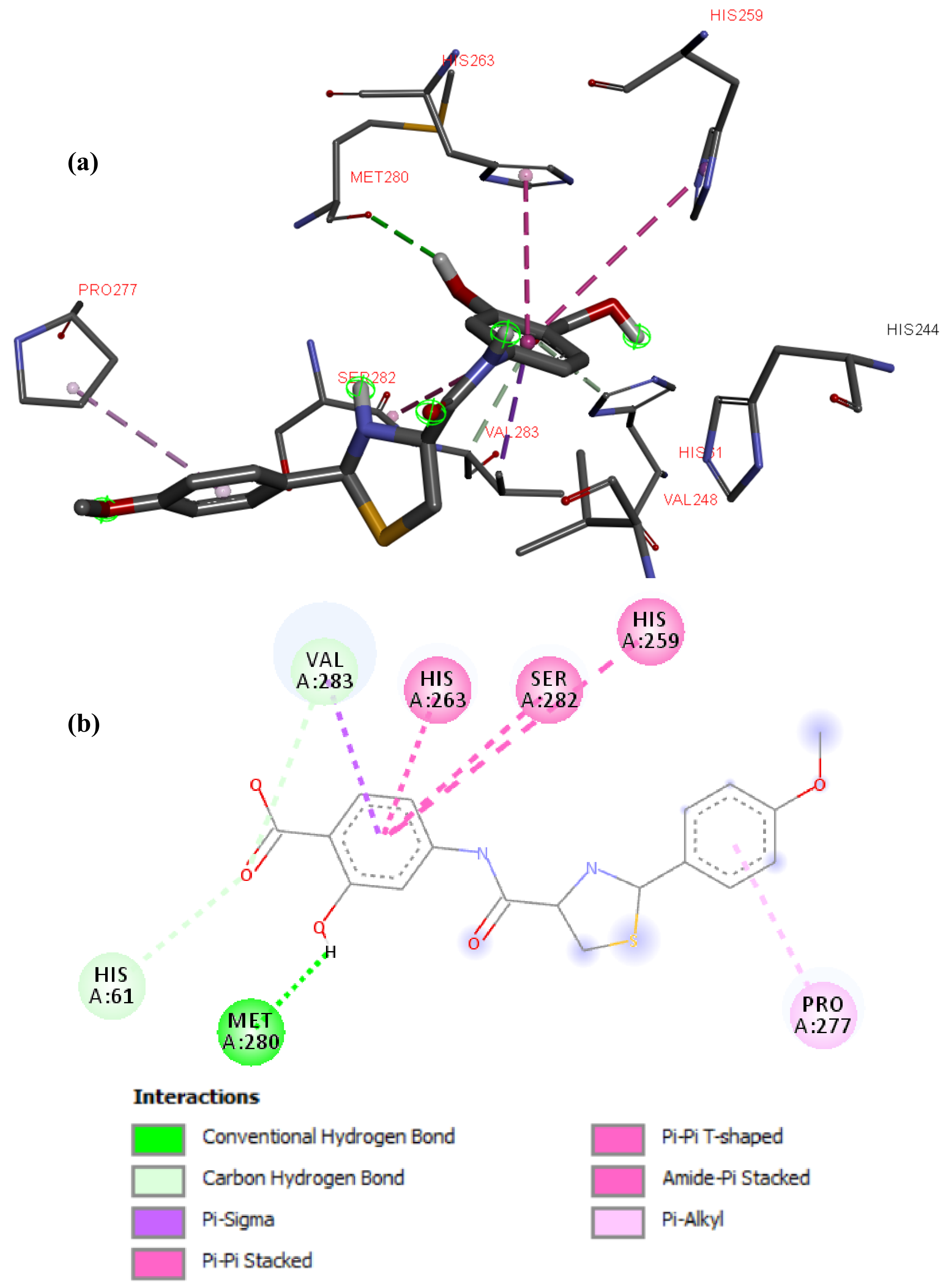

Figure S21. Binding interactions between derivative 4d and mushroom tyrosinase active site (PDB-ID 2Y9X). a) Binding conformation of the compound 4d in three-dimension along with the binding interaction of protein-ligand complex. b) Interaction patterns in 2D indicating the type and distance of the interaction. Legend indicates various types of binding interactions present.

(a)

(b)

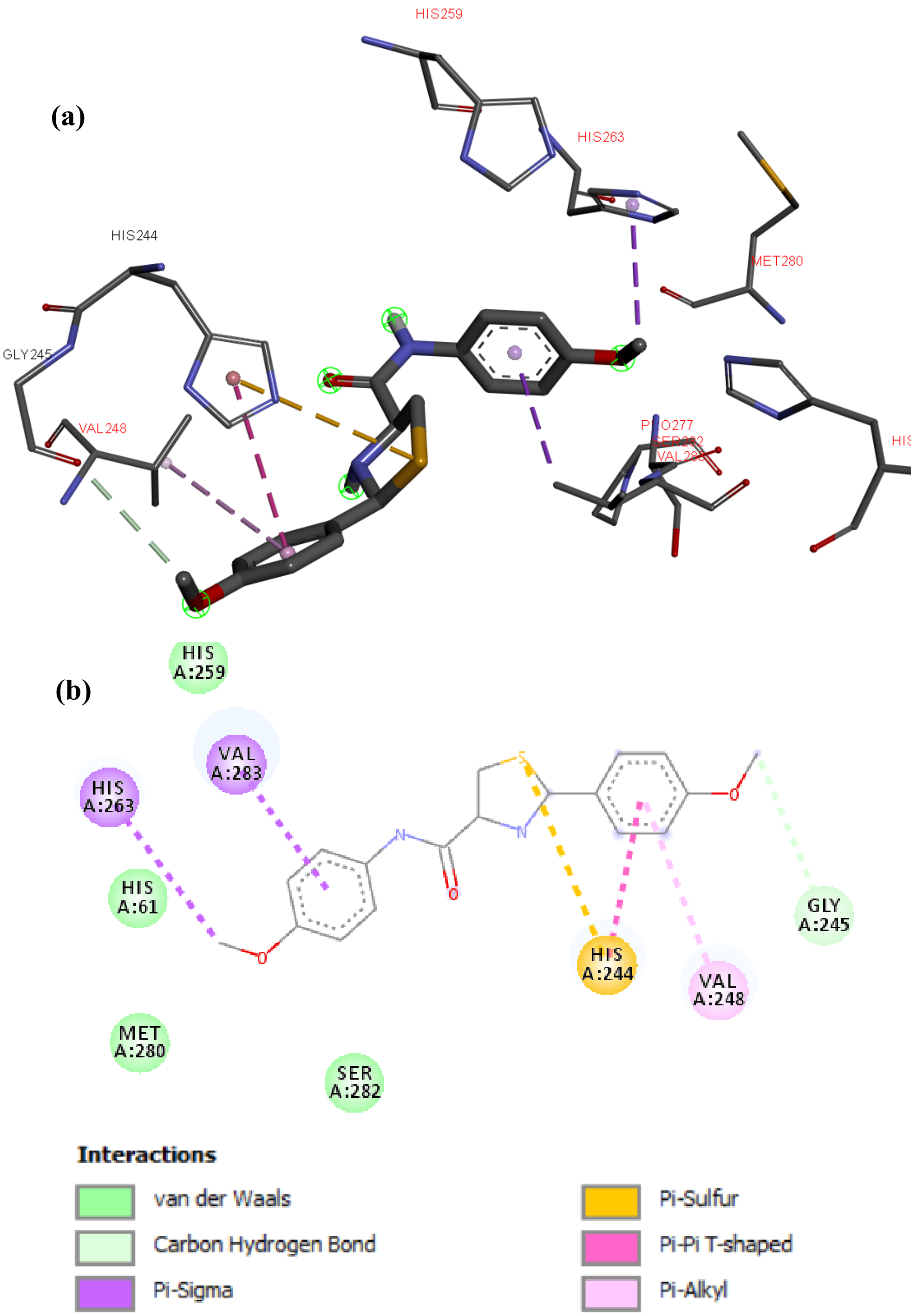

Figure S22. Binding interactions between derivative 4e and mushroom tyrosinase active site (PDB-ID 2Y9X). a) Binding conformation of the compound 4e in three-dimension along with the binding interaction of protein-ligand complex. b) Interaction patterns in 2D indicating the type and distance of the interaction. Legend indicates various types of binding interactions present.

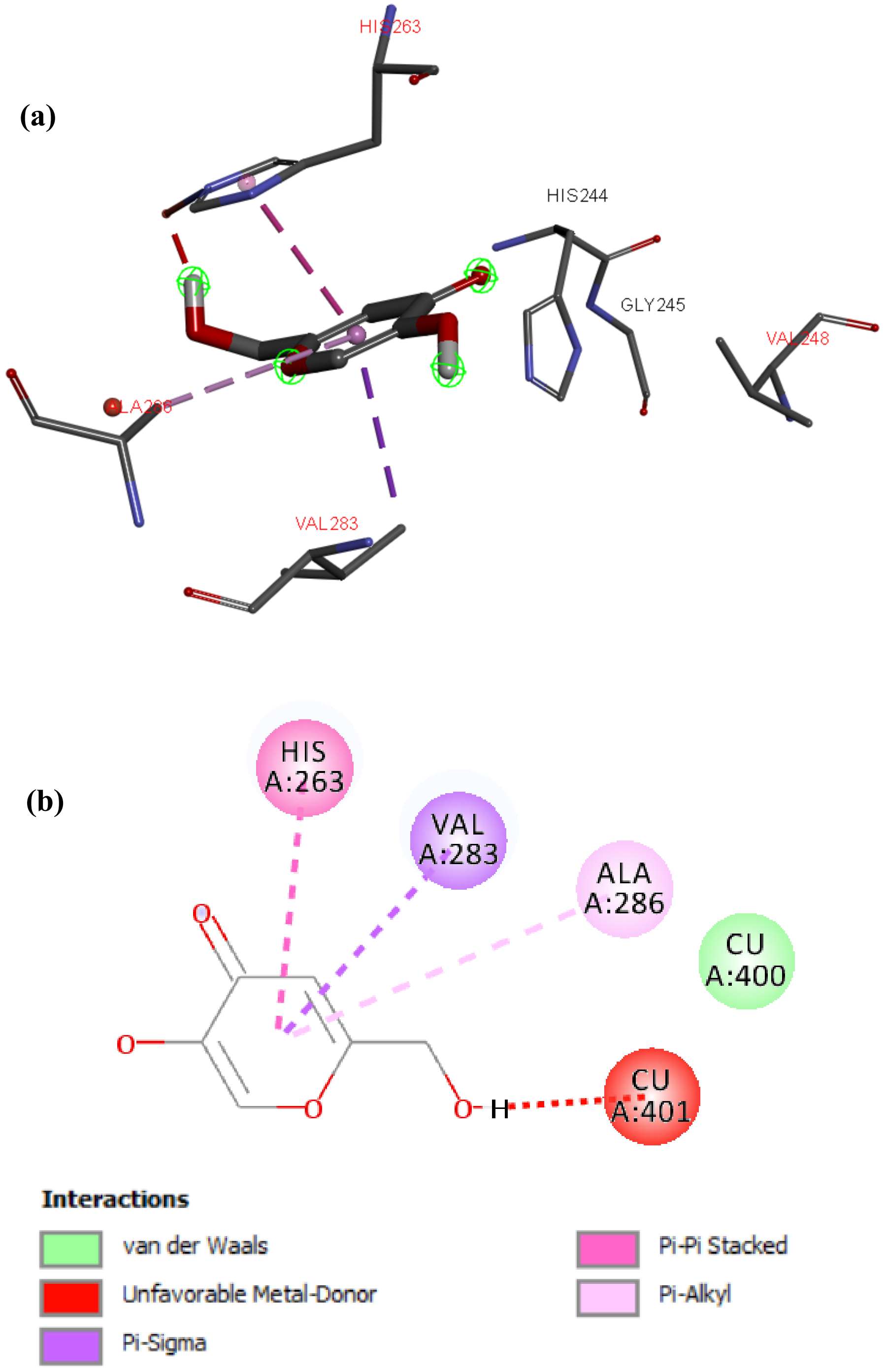

Figure S23. Binding interactions between kojic acid and mushroom tyrosinase active site (PDB-ID 2Y9X). a) Binding conformation of the compound kojic acid in three-dimension along with the binding interaction of protein-ligand complex. b) Interaction patterns in 2D indicating the type and distance of the interaction. Legend indicates various types of binding interactions present.
